# The ubiquitous pyridoxal 5′‐phosphate‐binding protein is also an RNA‐binding protein

**DOI:** 10.1002/pro.5242

**Published:** 2024-11-27

**Authors:** Claudio Graziani, Anna Barile, Alessia Parroni, Martino Luigi di Salvo, Irene De Cecio, Teresa Colombo, Jill Babor, Valérie de Crécy‐Lagard, Roberto Contestabile, Angela Tramonti

**Affiliations:** ^1^ Dipartimento di Scienze Biochimiche “A. Rossi Fanelli” Sapienza Università di Roma Rome Italy; ^2^ Istituto Pasteur Italy‐Fondazione Cenci Bolognetti Rome Italy; ^3^ Istituto di Biologia e Patologia Molecolari Consiglio Nazionale delle Ricerche Rome Italy; ^4^ Department of Microbiology and Cell Science University of Florida Gainesville Florida USA; ^5^ University of Florida Genetics Institute Gainesville Florida USA

**Keywords:** pyridoxal 5′‐phosphate, pyridoxal 5′‐phosphate homeostasis, pyridoxal 5′‐phosphate‐binding protein, RNA‐binding protein, vitamin B_6_ metabolism

## Abstract

The pyridoxal 5′‐phosphate binding protein (PLP‐BP) is believed to play a crucial role in PLP homeostasis, which may explain why it is found in living organisms from all kingdoms. *Escherichia coli* YggS is the most studied homolog, but human PLP‐BP has also attracted much attention because variants of this protein are responsible for a severe form of B_6_‐responsive neonatal epilepsy. Yet, how PLP‐BP is involved in PLP homeostasis, and thus what its actual function is in cellular metabolism, is entirely unknown. The present study shows that YggS binds RNA and that the strength of this interaction is modulated by PLP. A key role in RNA binding is clearly played by Lys137, an invariant residue located on a protein loop away from the PLP binding site, whose importance has been highlighted previously. The interaction with RNA is evidently conserved, since it is also observed with human PLP‐BP. The RNA binding site, which is apparently located at the entrance of the PLP‐binding site, is also evolutionarily conserved. It is therefore reasonable to assume that PLP, by defining the conformation of the protein, determines the RNA binding affinity. RNA‐seq analysis of RNA co‐purified with or captured by YggS revealed *SsrA* and *RnpB* RNAs, respectively involved in trans‐translation and tRNA maturation, as the major molecular components. This work opens up new horizons for the function of the PLP‐BP, which could be related to its interaction with RNA and modulated by PLP, and thus play a role in an as yet unknown regulatory mechanism.

## 1 INTRODUCTION

Although many aspects of vitamin B_6_ metabolism have been elucidated, fundamental questions remain unanswered. Perhaps, the most interesting and challenging one concerns the so‐called pyridoxal 5′‐phosphate binding protein (PLP‐BP). PLP, the catalytically active form of vitamin B_6_, is one of the six interconvertible B_6_ vitamers (pyridoxal, PL; pyridoxine, PN; pyridoxamine, PM; and their 5′‐phosphate forms PLP, PNP and PMP) and acts as a cofactor in many areas of metabolism (Percudani & Peracchi, 2003). Around 1.5% of prokaryotic genes encode PLP‐dependent enzymes, underlining the importance of this cofactor for microbial metabolism (Percudani & Peracchi, 2009). In humans, defects affecting PLP metabolism have been linked to many pathologies, particularly of the nervous system, and include vitamin B_6_‐dependent epilepsies (Wilson et al., 2019). *Escherichia coli*, like most prokaryotes and plants, is able to synthesize PLP de novo (Tramonti et al., 2021), whereas humans and other vertebrates acquire B_6_ vitamers from the diet and recycle them into PLP via a salvage pathway (di Salvo et al., 2011, 2012).

Among all the characters involved in vitamin B_6_ metabolism, a ubiquitous protein has recently emerged from research in this field. This protein binds PLP very tightly via a classical Schiff base with a conserved lysine residue, and plays an important role in PLP homeostasis, although it appears to lack catalytic activity (Eswaramoorthy et al., 2003; Ito et al., 2013). As its function is not yet understood, this protein has been given the generic name of PLP‐BP. PLP‐BP was named YggS in *E. coli* (Ito et al., 2013) and PROSC in humans (Prunetti et al., 2016), and is a member of the COG0325 family. Various pleiotropic phenotypes of the *E. coli yggS* knockout mutant strain have been reported, which are clearly linked to amino acid and vitamin B_6_ metabolism and reflect an imbalance in PLP homeostasis (Ito, 2022). The *E. coli yggS* knockout mutant accumulates the PLP precursor pyridoxine phosphate (PNP) and is sensitive to an excess of pyridoxine but not of pyridoxal. The pyridoxine toxicity phenotype is complemented by the expression of eukaryotic *yggS* orthologues, including human PLP‐BP (Prunetti et al., 2016). The phenotype is also suppressed by the presence of amino acids, specifically isoleucine, threonine and leucine, suggesting that PLP‐dependent enzymes are affected (Ito et al., 2013). However, the actual role of YggS in PLP homeostasis has not been understood. Importantly, mutations of the gene encoding PLP‐BP in humans are responsible for a rare and severe form of B_6_‐responsive epilepsy (Darin et al., 2016; Johnstone et al., 2019; Plecko et al., 2017; Shiraku et al., 2018). This is taken as clear evidence of the involvement of PLP‐BP in PLP homeostasis, since several PLP‐dependent enzymes are involved in neurotransmitter metabolism. A recent analysis has shown that while most kingdoms have a single PLP‐BP homolog, Angiosperms within the plant kingdom have two. In *Arabidopsis thaliana*, where these are annotated as PLP homeostasis proteins, they are important for management of PLP (Farkas & Fitzpatrick, 2024). The crystal structures of the yeast (Eswaramoorthy et al., 2003) and *E. coli* proteins (PDB codes: 1CT5 and 1W8G, respectively) have been determined. Like alanine racemase or ornithine decarboxylase (PLP‐dependent enzymes), the proteins fold as a TIM barrel (Eswaramoorthy et al., 2003). However, unlike these enzymes that are two‐domain homodimers, PLP‐BP is a single‐domain monomer (Tramonti et al., 2022), consisting exclusively of the barrel domain, and shows a conserved and solvent exposed PLP binding site.

Despite the large amount of published research on PLP‐BP, no specific proposal for its function has yet been experimentally proven. The most popular hypothesis concerns a possible role as PLP transporter and distributor (Darin et al., 2016; Prunetti et al., 2016). According to this hypothesis, PLP‐BP could be a shuttle protein that brings PLP to target enzymes through a direct channeling mechanism, in which a complex is formed between PLP‐BP and PLP‐dependent apoenzymes. PLP‐BP may protect PLP from the environment, sequestering it and at the same time constituting a reservoir of the cofactor for PLP‐enzymes that might tap into. However, YggS has a very high affinity for PLP (whose binding is characterized by a large *k*_on_ and a small *k*_off_) and it is able to transfer PLP to PLP‐dependent apoenzymes with very slow transfer kinetics (Tramonti et al., 2022). Moreover, it is not among the most abundant proteins in the *E. coli* cell (PaxDb: Protein Abundance Database; https://pax-db.org/protein/511145/b2779). Therefore, this hypothesis does not seem plausible, not least because PLP‐dependent enzymes have very different structures, and it is thus unlikely that they could all be recognized by the same protein. Moreover, the observation that an *yggS E. coli* deletion strain grows normally (Babor et al., 2023; Prunetti et al., 2016) is inconsistent with the hypothesis that YggS serves a fundamental function as a PLP transporter. On the other hand, the presence in YggS of a conserved cluster of positively charged lysine residues suggests that the protein may interact with negatively charged regions of other macromolecules (Tramonti et al., 2022). In particular, results obtained in in vivo experiments showed that the K137A variant (K137 is part of the lysine cluster and is located in a surface polypeptide loop far from the PLP‐binding site), while having no effect on PLP binding, does not complement a phenotype observed with the *yggS* deletion strain, which is the increased sensitivity to the pyridoxine analogue 4‐deoxypyridoxine. This observation suggests that K137 may be involved in interactions with other macromolecules and envisages for YggS a role as component of a regulatory pathway (Tramonti et al., 2022).

Therefore, following the hypothesis that YggS might be a protein that senses PLP levels and acts as part of a regulatory mechanism, we decided and tried to identify its possible interactors. The results we obtained clearly indicate that the protein specifically binds RNA.

## 2 RESULTS

### 2.1 Identification of *Escherichia coli* PLP‐BP (YggS) interactors

**FIGURE 1:**
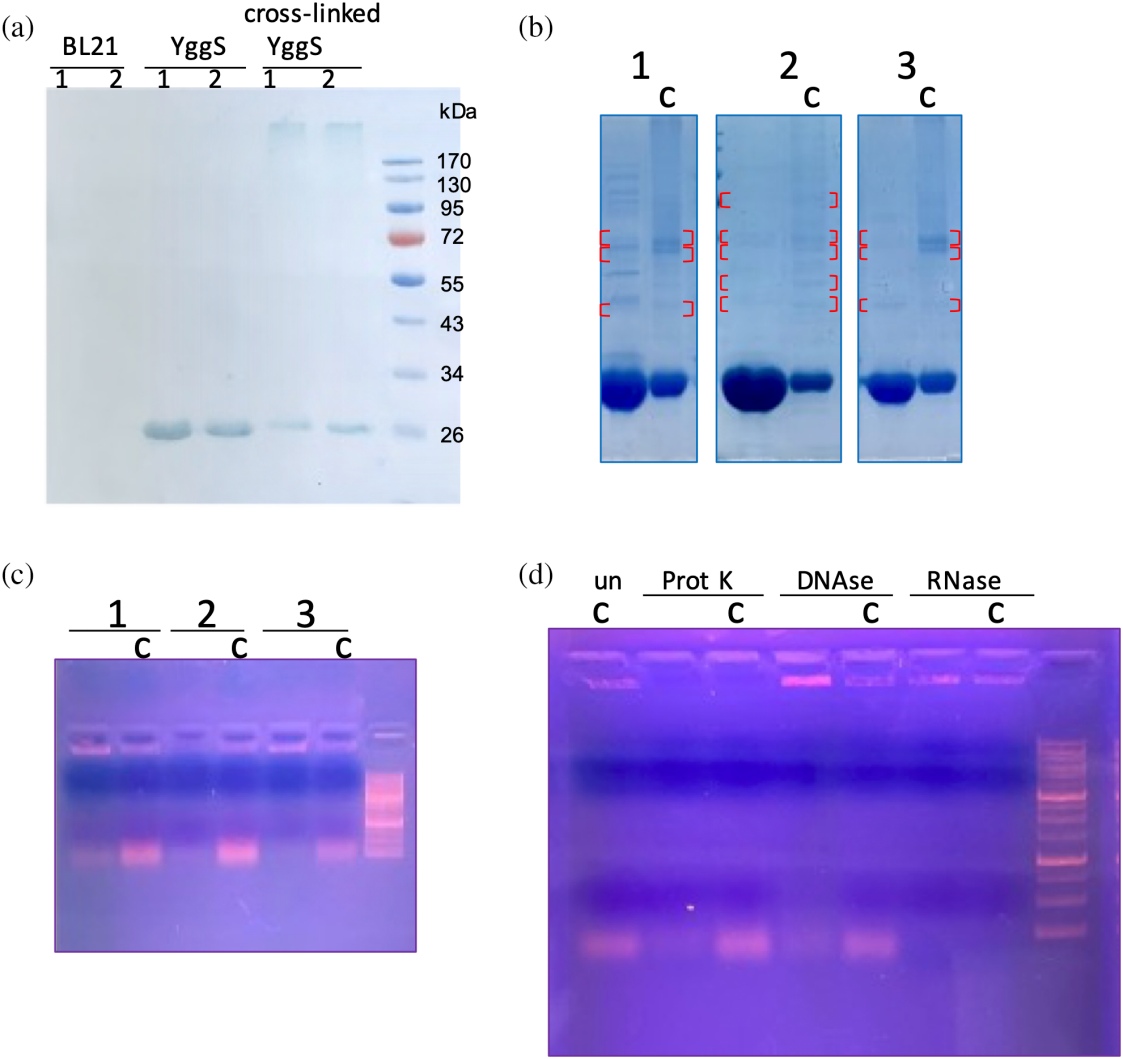
Results of formaldehyde cross‐linking experiments. (a) Cell extracts (in biological duplicate) from BL21(DE3) *E. coli* cells (BL21) and from BL21(DE3)/pET28*yggS* cells overexpressing YggS, untreated (YggS) or treated with formaldehyde (cross‐linked YggS), were subjected to SDS‐PAGE and subsequent Western blot analysis using anti‐polyHistidines antibody; the molecular weight marker is the PageRuler™ Prestained Protein Ladder (Thermo Scientific). A protein band with a molecular weight corresponding to that of YggS is only visible in the lanes of extracts obtained from cells overexpressing the protein. It can be noticed that the electrophoretic mobility of YggS is slightly lower in the cross‐linked samples. (b) SDS‐PAGE analysis of three consecutive fractions (1, 2 and 3) from NiNTA purification of YggS, expressed in formaldehyde‐treated (c) and untreated cells, containing the highest protein concentration. YggS is visible in all lanes as a very intense protein band. The higher molecular weight bands visible in the lanes of the cross‐linked samples and in the corresponding gel zones in the lanes of the non‐cross‐linked samples were analyzed by mass spectrometry. The areas of the gel excised and analyzed by mass spectrometry are indicated by red square brackets. (c) Agarose gel electrophoresis analysis of the same fractions (1, 2 and 3) of Ni‐NTA purified YggS shown in panel B together with the 1Kb plus DNA ladder (Thermo Scientific). In addition to the low‐molecular‐weight nucleic acid band, a high‐molecular‐weight band is also visible, especially in the not cross‐linked sample 1 (lane 1). (d) Agarose gel electrophoresis analysis of samples from fraction 1, shown in panels B and C, untreated (un) and after incubation with proteinase K, DNase I (RNase free) and RNase I. Treatment with proteinase K and RNase causes the disappearance of the high‐molecular‐weight band, demonstrating that this corresponds to a protein–RNA complex.

**FIGURE 2:**
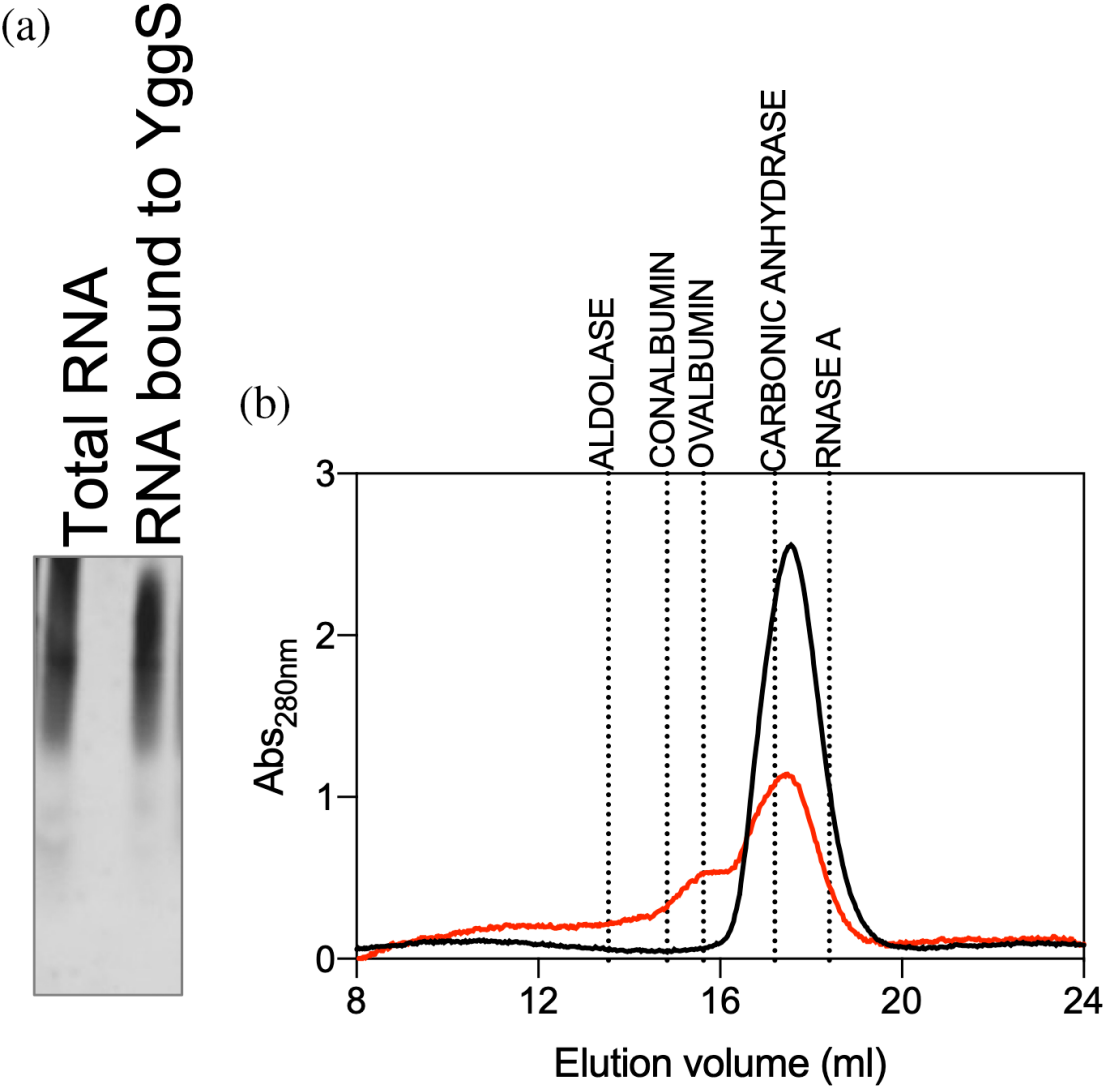
Analysis of the RNA‐YggS complex isolated from cross‐linking experiments. (a) Urea denaturing PAGE comparison of total RNA extracted from *E. coli* BW25113 cells and RNA cross‐linked to YggS. (b) Size Exclusion Chromatography analysis of purified cross‐linked YggS (red line), compared to YggS purified from cells than were not treated with formaldehyde (black line). The chromatogram shows absorbance at 280 nm as a function of elution volume. The elution volumes of the MW standards used to calibrate the column are indicated: aldolase (158 kDa, 13.54 mL), conalbumin (75 kDa, 14.83 mL), ovalbumin (44 kDa, 15.63 mL), carbonic anhydrase (29 kDa, 17.2 mL), and RNase A (17.7 kDa, 18.4 mL).

To identify possible PLP‐BP interactors, we performed in vivo cross‐linking experiments in *E. coli* cells overexpressing recombinant YggS. We first used formaldehyde as a cross‐linking reagent. Western blot analysis of the bacterial lysates revealed that the cross‐linked YggS has a slightly reduced electrophoretic mobility, evidently resulting from chemical conjugation; in addition, a high molecular weight band is visible (Figure 1a). The protein was purified by Ni‐NTA affinity chromatography from bacterial cells that were either treated or not with the cross‐linking reagent, and then analyzed by SDS‐PAGE. High molecular weight bands resulting from the electrophoresis of different chromatography fractions (Figure 1b) were excised and then analyzed by mass spectrometry to identify proteins conjugated with YggS. However, the analysis did not reveal the presence of any proteins other than YggS (data not shown), showing that the high molecular weight bands resulted from the conjugation of multiple molecules of YggS itself. At this point, fractions collected during YggS purification by Ni‐NTA chromatography were analyzed by agarose gel electrophoresis and staining with a fluorescent intercalating agent (GelRed; Merck), revealing the presence of nucleic acids (Figure 1c). After digestion with proteinase K, DNase and RNase I, it was clear that the conjugated material was RNA, and that this was present in YggS samples obtained from both formaldehyde‐treated and untreated cells, although to a greater extent in the former (Figure 1d). The RNA extracted from the cross‐linked protein samples and analyzed on a denaturing urea PAGE is heterogeneous. However, in terms of molecular weight assortment, it is somewhat different from a total RNA sample extracted from *E. coli* cells, showing that YggS binds only a subset of RNA molecules (Figure 2a). The size exclusion chromatography analysis of the YggS samples purified from formaldehyde‐treated and untreated cells revealed that, in the cross‐linked samples, YggS was present both in the monomeric form (Figure 2b, elution volume 16–20 mL) and in a larger size form (Figure 2b, elution volume 12–16 mL), which evidently corresponds to an RNA‐conjugated form, as demonstrated by agarose gel electrophoresis and staining with GelRed (Figure S1).

**FIGURE 3:**
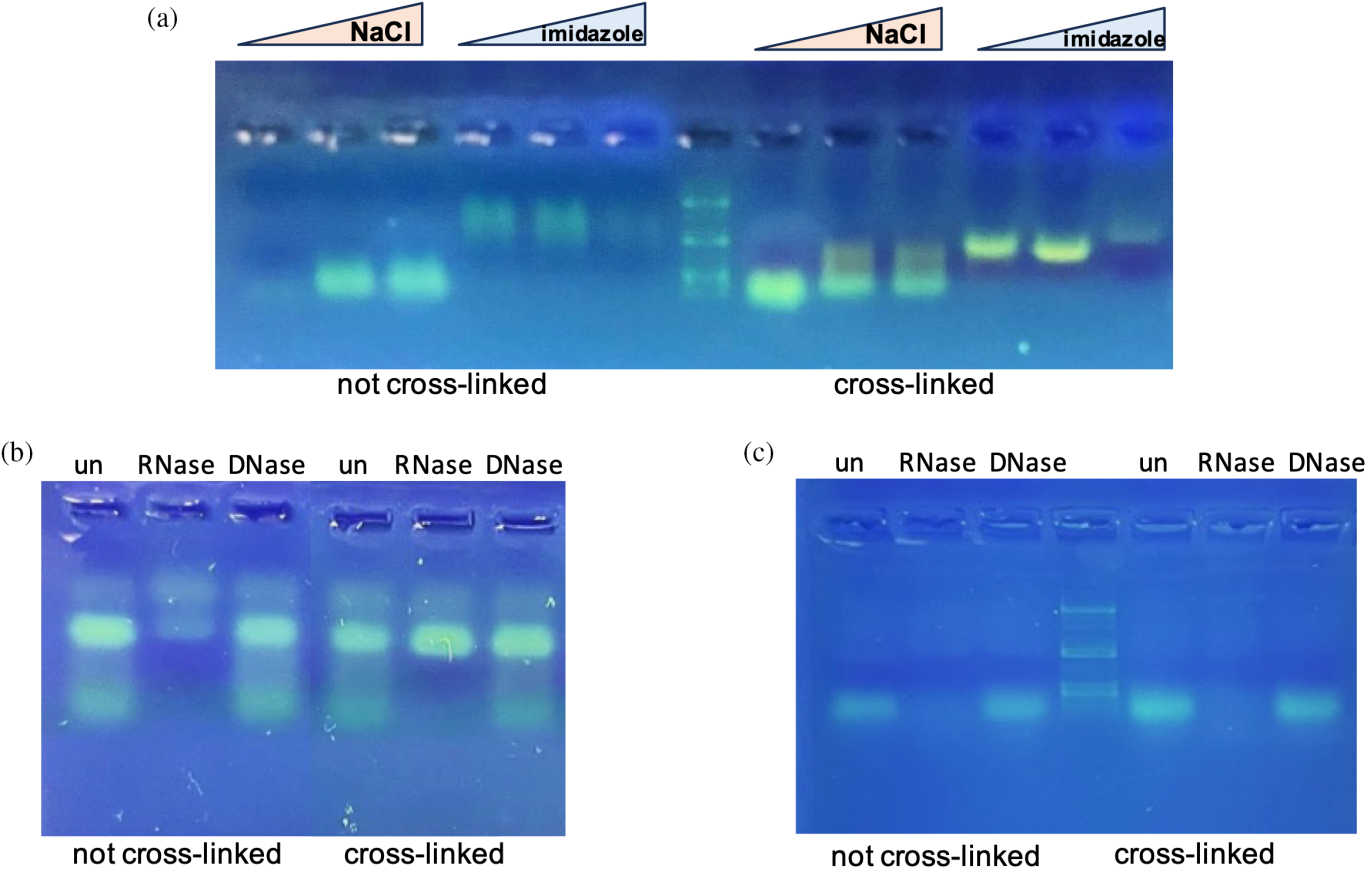
Results of UV cross‐linking experiments. (a) Agarose gel electrophoresis analysis of Ni‐NTA chromatography fractions obtained after elution with NaCl and imidazole of extracts from *E. coli* cells that were subjected to UV cross‐linking or not. (b) Analysis of the effects of RNase I and DNase I (RNase free) digestion of nucleic acid collected in the fractions eluted with imidazole. Note that the nucleic acid is not digested by DNase and is only partially digested by RNase I. This is most evident in samples that were subjected to UV cross‐linking. (c) When the same samples were treated with proteinase K prior to digestion with RNase I, the nucleic acid was completely digested, showing that the association with the protein protects RNA from digestion. The 1Kb plus DNA ladder was used as a standard.

Given the evidence of RNA binding to YggS, we performed in vivo UV cross‐linking, which is more specific for RNA than formaldehyde and it induces the formation of covalent bonds between the protein and components that are in direct contact with it at the instant of irradiation (Urdaneta & Beckmann, 2020). *E. coli* cells overexpressing YggS were either exposed or not to UV radiation (*λ* = 254 nm). The cell lysate was loaded onto a Ni‐NTA column, washed with the equilibration buffer to eliminate any unbound material, firstly eluted with a NaCl gradient (0 → 1 M), to get rid of nucleic acids that were either weakly or non‐covalently bound to protein, and then eluted with an imidazole gradient (0 → 300 mM) to elute the protein with the nucleic acid possibly bound to it. SDS‐PAGE analysis of the collected fractions showed that, with both UV treated and untreated samples, the protein eluted from the column only following imidazole treatment (data not shown). Instead, weakly bound nucleic acid was eluted with NaCl gradient, while RNA–protein complexes were eluted with imidazole gradient (Figure 3a). Analysis by agarose gel electrophoresis shows that the nucleic acid component is different in the fractions eluted with NaCl than in those eluted with imidazole, and there are also differences between UV‐treated and untreated samples. In particular, the nucleic acid molecules present in the fractions eluted with NaCl are of lower molecular weight than those eluted with imidazole. The nucleic acid bands from UV‐treated cells are particularly intense and sharp in the fractions eluted with imidazole. Fractions eluted with imidazole were collected, washed to eliminate imidazole and concentrated. Samples were then subjected to RNase I and DNase digestion, demonstrating that the nucleic acid associated with the protein is RNA and that in the UV‐cross‐linked sample the protein effectively protects RNA from digestion by RNase I (Figure 3b). To check this latter point, all samples were treated with proteinase K prior to digestion with RNase I (Figure 3c): as expected, the complete degradation of RNA was observed.

### 2.2 Analysis of RNA binding to wild type and variant forms of YggS

In order to study the protein structural features involved in RNA binding, we used variant forms of YggS. We focused our attention on two lysine residues that are proposed to be involved in YggS function: Lys36 and Lys137. Both residues are part of a cluster of positively charged residues that affects YggS stability and conformation and plays an important role in its function (Tramonti et al., 2022). It is worth mentioning that an *E. coli yggS* deletion strain exhibits a concentration‐dependent growth sensitivity phenotype to the pyridoxine analogue 4‐deoxypyridoxine (Babor et al., 2023). This phenotype is complemented by the expression of wild type YggS *in trans*, whereas it is not complemented by the expression of YggS K36A and K137A variants, showing that both lysine residues are functionally important. The K36 residue is responsible for the covalent binding of PLP at the canonical PLP‐binding site (notice that the K36A variant is still able to bind PLP covalently, but at a different site; Tramonti et al., 2022), whereas Lys137, which has no influence at all on PLP binding and is located on a solvent‐exposed peptide loop of the protein, may possibly be involved in the interaction with other macromolecules (Tramonti et al., 2022).

**FIGURE 4:**
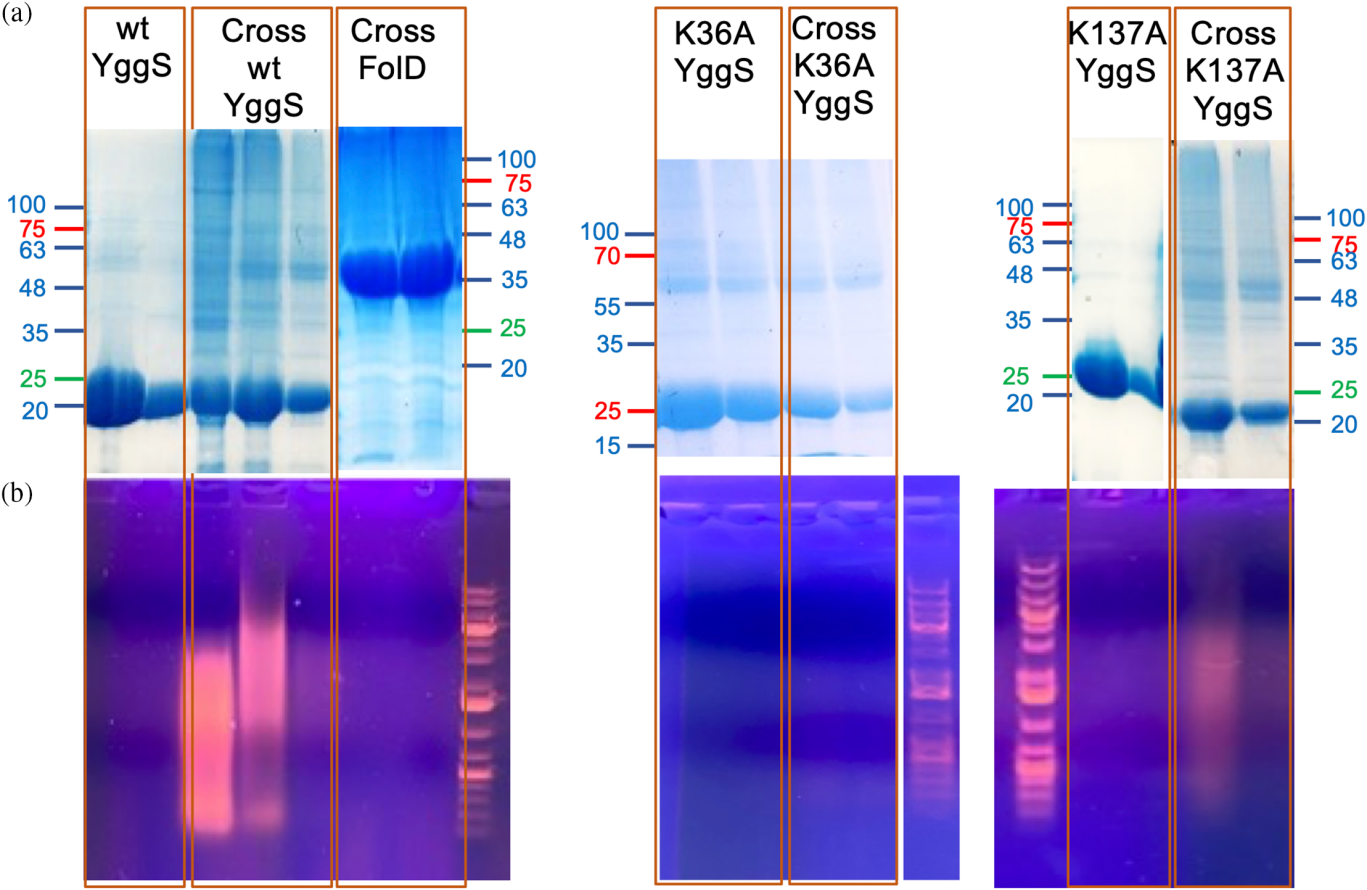
SDS‐PAGE (upper panels, a) and agarose gel electrophoresis (lower panels, b) analyses of wild type and variant forms (K36A and K137A) of YggS purified from formaldehyde‐treated and untreated cells. Only fractions from Ni‐NTA chromatography containing the highest protein concentration were analyzed. As a control, fractions from purification of FolD expressed in formaldehyde‐treated cells were analyzed. For clarity, in SDS‐PAGE analyses, only the positions corresponding to molecular weight standards are indicated (PageRuler Plus Prestained Protein Ladder, Thermo Fisher Scientific, for K36A and Opti‐Protein XL Marker, Applied Biological Materials Inc., Richmond, Canada, for WT, K137A and FolD), whereas in the agarose gel electrophoreses the 1Kb plus DNA ladder standard is visible.

Fractions collected from Ni‐NTA affinity chromatography during the purification of K36A and K137A variant proteins, expressed in cells that were either treated or not with formaldehyde, were analyzed by both SDS‐PAGE and agarose gel electrophoresis (Figure 4a,b). YggS K36A and K137A samples contained a significantly lower amount of co‐purified RNA compared to wild type YggS, suggesting a lower binding affinity of these variant forms for RNA. As a negative control, *E. coli* FolD, an enzyme which is involved in folate metabolism, was expressed and purified following the same procedures as YggS. Agarose gel electrophoresis (Figure 4b) of Ni‐NTA FolD fractions purified from cells treated with formaldehyde showed no detectable co‐purified RNA.

**FIGURE 5:**
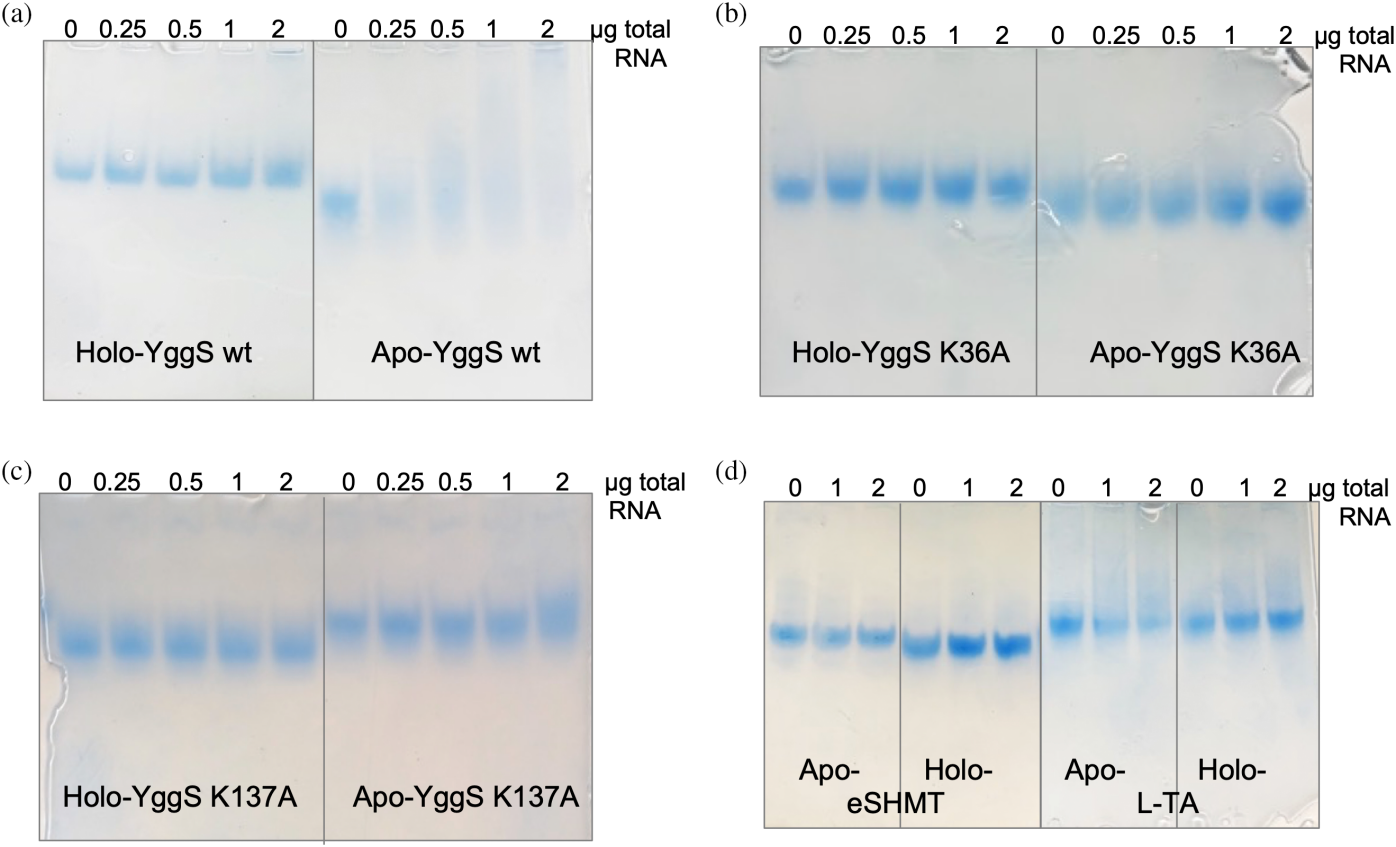
REMSA analysis of RNA binding to YggS using total RNA. REMSA analyses were carried out with purified recombinant wild type (a), K36A (b) and K137A (c) YggS variants in the apo‐ and holo‐forms (1 μg) incubated with increasing amounts (0.25, 0.5, 1 and 2 μg) of total RNA extracted from BW25113 *E. coli* cells. As a control, purified apo‐ and holo‐*e*SHMT and L‐TA were also analyzed in the same conditions used for YggS (d). Native TBE 5% polyacrylamide gels were stained with Quick Coomassie dye.

REMSA (RNA electrophoretic mobility shift assay) analyses were then carried out on purified recombinant wild type and variant YggS proteins (1 μg) incubated with increasing amounts of total RNA extracted from BW25113 *E. coli* cells. To ensure that the proteins used in REMSA did not have nucleic acids already bound, RNase I and high ionic strength buffer treatments were performed during purification (see Section 4). Both the holo‐form (containing PLP) and the apo‐form of the proteins were used in the assays. The results clearly show that RNA binds with much higher affinity to the apo‐form than to the holo‐form of wild type YggS (Figure 5a). On the other hand, both K36A and K137A variants do not show any RNA binding, neither in the holo‐form nor in the apo‐form (Figure 5b,c). Two different *E. coli* PLP‐dependent enzymes, serine hydroxymethyltransferase (*e*SHMT) and L‐threonine aldolase (L‐TA), were also used in the analyses as negative controls, showing that *E. coli* total RNA does not bind to these proteins (Figure 5d). Moreover, to exclude the possibility that the binding of RNA to YggS was affected by the 6x histidine tag that is present on the recombinantly expressed protein, the tag was removed from the N‐terminal end of YggS, and this form was compared to the tagged form of YggS. No difference between the two protein forms, concerning RNA binding, was observed (Figure S2).

**FIGURE 6:**
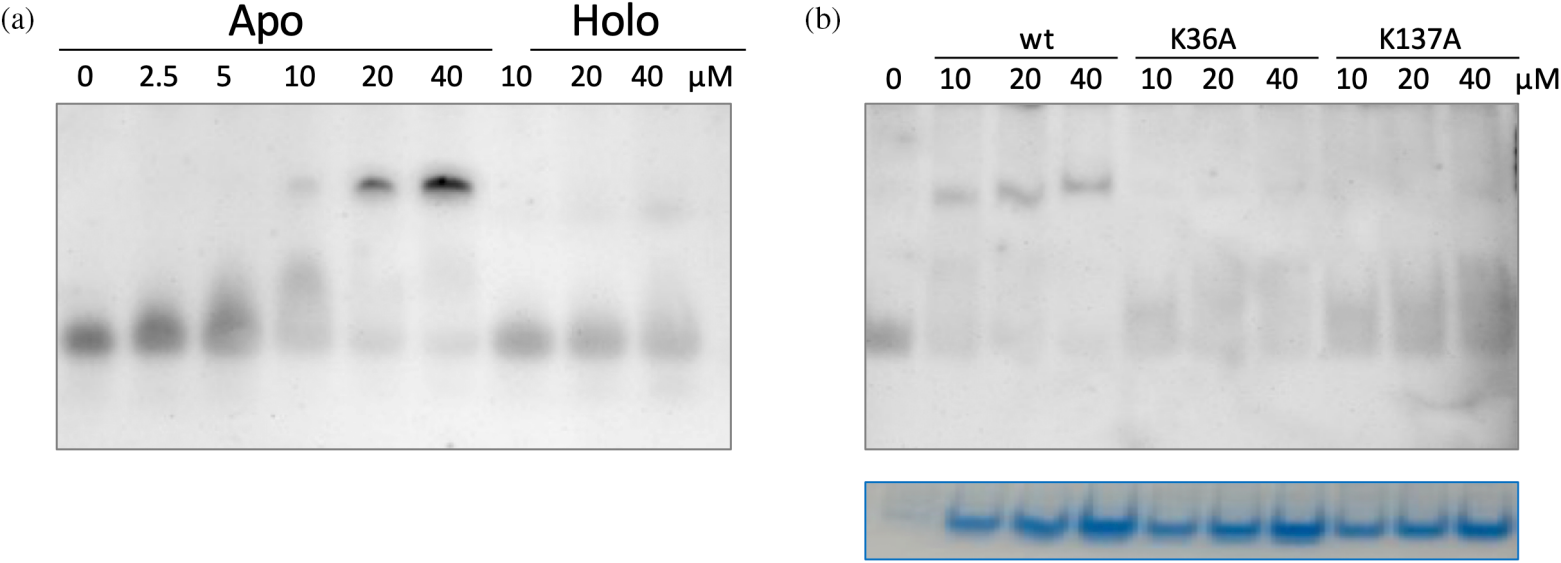
REMSA analysis of RNA binding to YggS using tRNA. (a) REMSA analyses carried out with tRNA (20 ng) incubated with increasing amounts of purified WT apo‐ and holo‐YggS (2.5, 5, 10, 20 and 40 μM). Naked tRNA and YggS–tRNA complexes were detected by staining with SyBr Green. (b) REMSA analysis carried out with tRNA (20 ng) incubated with increasing amounts of purified wild type, K36A and K137A apo‐YggS forms (10, 20 and 40 μM). The same native TBE 5% polyacrylamide gel was first stained with SyBr Green to visualize the tRNA and the tRNA–protein complexes (upper panel), and then with Quick Coomassie dye to check the amount of different protein forms (lower panel).

In the REMSA analyses shown above, total *E. coli* RNA was used, that is, containing all major forms of RNA (transfer, messenger and ribosomal RNA). However, low molecular weight RNA from *E. coli* BW25113 cells, consisting mainly of tRNA, was also purified and used in REMSA analyses with increasing amounts of the different forms of YggS. tRNA is visible as a focused band in REMSA, allowing a much clearer analysis of binding to YggS (Figure 6). In the analyses using WT YggS, the formation of a slowly migrating band corresponding to a protein–tRNA complex is clearly visible, whose density increases as the protein concentration is increased. With apo‐YggS, densitometric measurements of the faster band (free tRNA) and the slower band (protein–tRNA complex) in four independent REMSA analyses allowed the estimate of an apparent *K*_d_ of 5.1 ± 0.3 μM. The addition of PLP (in a 5‐fold stoichiometric ratio with respect to the apo‐protein) negatively affects the formation of the protein–RNA complex, giving an apparent *K*_d_ of 23 ± 5 μM (Figure 6a). When the two K36A and K137A YggS variants, in either the apo‐ or holo‐forms, were used, no definite bands attributable to a YggS–tRNA complex could be observed (Figure 6b). Other proteins, such as *e*SHMT and L‐TA, did not bind tRNA in vitro (Figure S3).

**FIGURE 7:**
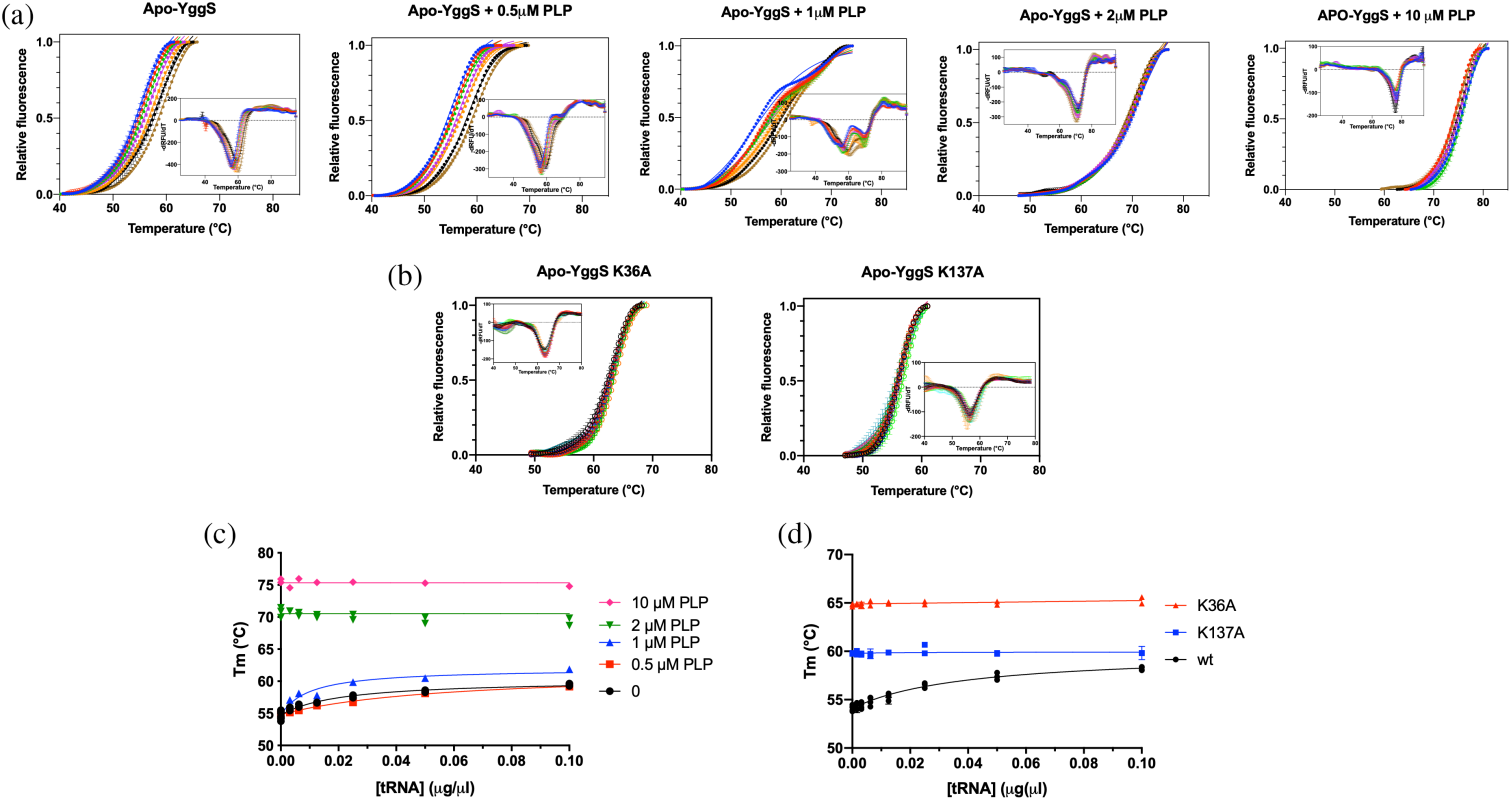
Differential scanning fluorimetry (DSF) analysis of tRNA binding to YggS. (a) DSF measurements of wild type apo‐YggS (2 μM) in the presence of increasing concentrations of tRNA (from 0 and 0.1 μg/μL) and of the indicated constant concentrations of PLP. Data are shown as fluorescence change expressed as fractional variation as a function of temperature (denaturation profiles). Data were fitted to the Boltzmann equation, as described in (Tramonti et al., 2022), to obtain apparent melting temperatures. Each curve is the average of three independent experiments. Insets are the first derivative (−d*F*/d*T*) of denaturation profiles. (b) DSF measurements of the K36A and K137A apo‐YggS variants carried out in the same conditions, in the absence of PLP. (c) Graph reporting the values of apparent melting temperature (*T*_m_) determined for wild type apo‐YggS as a function of tRNA concentration at the indicated PLP concentration. The continuous lines through data result from fitting to a hyperbolic saturation equation. (d) Graph of *T*_m_ as a function of tRNA concentration for the WT, K36A and K137A variants in the absence of PLP.

We have previously shown that binding of PLP to apo‐YggS has a large stabilizing effect on the protein, which is evident in the increase of the apparent melting temperature (*T*_m_). This effect is due to the stabilization of Lys36, which may be considered as a frustrating residue, and is accompanied by a conformational change of the protein (Tramonti et al., 2022). In fact, the apo‐form of the K36A variant has a high *T*_m_, which is not increased upon PLP binding. This is explained by apo‐K36A having a holo‐like conformation. On the contrary, the *T*_m_ of apo‐K137A, which is a bit higher than apo‐WT, is increased by PLP binding, showing that a conformational change takes place upon binding of the cofactor (Tramonti et al., 2022). Here we used the same technique (differential scanning fluorimetry, DSF) to analyze the effect of tRNA binding on the thermal stability of WT and variant forms of apo‐YggS. Figure 7a shows that tRNA increases the thermal stability of WT apo‐YggS. However, if the protein is saturated with PLP, the *T*_m_, which is already greatly increased by PLP binding, does not change further when tRNA is added (Figure 7c). Taken together with the results of REMSA analyses, this observation indicates that PLP and tRNA binding are mutually exclusive options for WT apo‐YggS. When the apo‐forms of K36A and K137A variants were incubated with increasing concentrations of tRNA, their thermal stability stayed constant (Figure 7b,d). This result suggests that the K36A variant does not bind RNA because it is in a holo‐like conformation, whereas the K137A variant, despite having an apo‐like conformation, is unable to bind RNA because the Lys137 residue is directly involved in this binding.

### 2.3 RNA‐binding properties of human PLP‐BP

**FIGURE 8:**
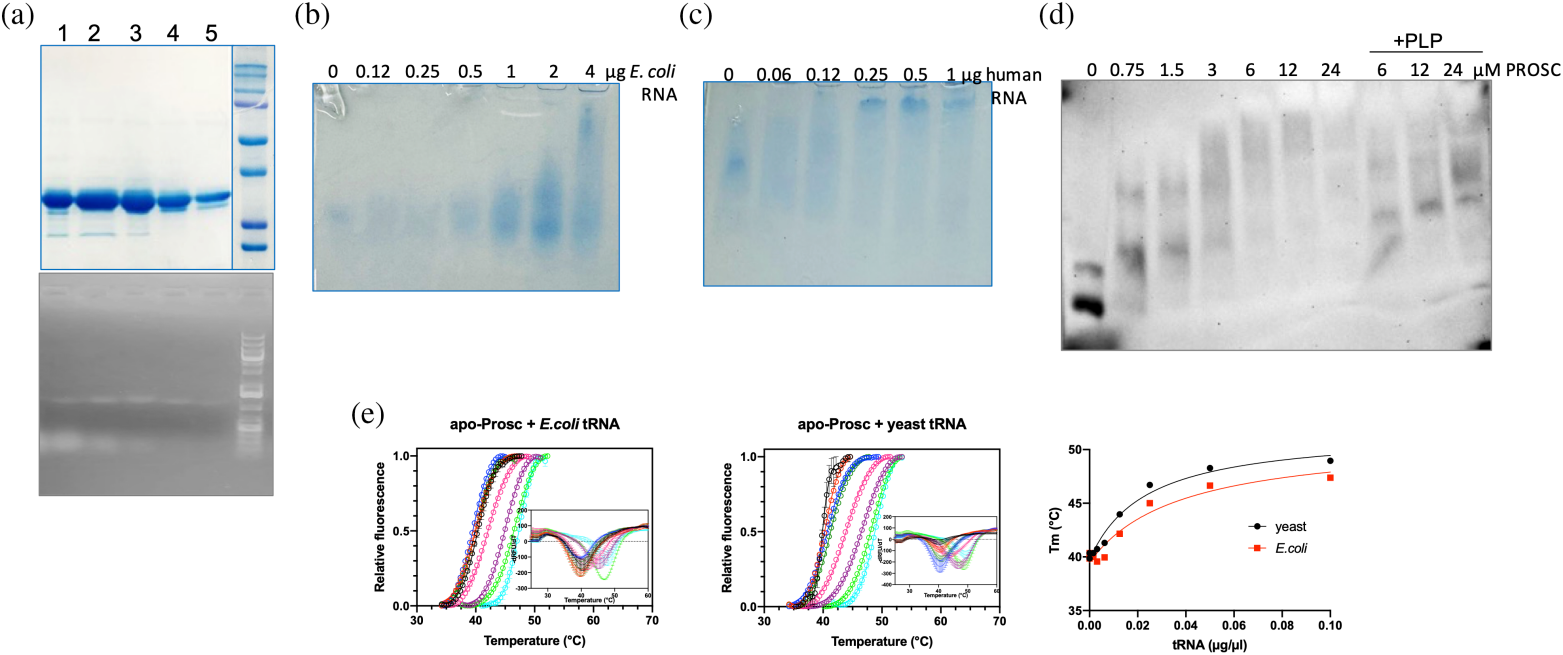
Analysis of RNA binding to human apo‐PROSC. (a) SDS‐PAGE (upper panel) and agarose gel electrophoresis (lower panel) analyses of PROSC purified from *E. coli* cells overexpressing the protein. Only fractions from Ni‐NTA chromatography containing the highest protein concentration were analyzed. (b) REMSA analysis using increasing concentrations of *E. coli* total RNA and a fixed amount of apo‐PROSC (1 μg). (c) REMSA analysis using increasing concentrations of human total RNA (extracted from MDA‐MB‐231 cells) and a fixed amount of apo‐PROSC (1 μg). Very similar results were obtained using RNA extracted from HAP1 cells. (d) REMSA analysis carried out with biotinylated *E. coli* tRNA (0.004 pmoles) incubated with increasing amounts of purified apo‐PROSC (from 0 to 24 μM). The last three lanes show samples of *E. coli* tRNA that were incubated with apo‐PROSC (from 6 to 24 μM) in the presence of 100 μM PLP, in order to obtain the holo‐protein. (e) DSF measurements of apo‐PROSC (2 μM) in the presence of increasing concentrations of either *E. coli* or yeast tRNA (from 0 and 0.1 μg/μL). Data are shown as fluorescence change expressed as fractional variation as a function of temperature (denaturation profiles). Data were fitted to the Boltzmann equation to obtain apparent melting temperatures. Each curve is the average of three independent experiments. Insets are the first derivative (−d*F*/d*T*) of denaturation profiles. In the graph reporting the values of apparent melting temperature (*T*_m_) determined as a function of tRNA, the continuous lines through data result from fitting to a hyperbolic saturation equation.

We recombinantly expressed PROSC, the human PLP‐BP, in *E. coli* and purified it to homogeneity. Differently from what reported by Fux and Sieber (2020), in our hands PROSC was almost exclusively in the monomeric form, although a minor fraction of the dimeric protein could be detected (Figure S4). Interestingly, this preparation of PROSC (purified by Ni‐NTA affinity chromatography as overexpressed recombinant protein) also contained nucleic acid, as shown by agarose gel electrophoresis and GelRed staining of chromatography fractions (Figure 8a). Again, the nucleic acid co‐purified with the protein turned out to be RNA, due to its sensitivity to RNase I but not to DNase (Figure S5). We observed that also *A. thaliana* PLP‐BP (isoform 1, At1g11930/F12F1_20), recombinantly expressed in *E. coli*, is co‐purified with RNA (data not shown). RNA binding by conserved proteins across different species provides compelling evidence that this property has a functional significance (Esteban‐Serna et al., 2023). Therefore, our results obtained with distantly related PLP‐BP homologs strongly suggest that the ability to bind RNA is a common feature of PLP‐BPs.

REMSA analyses, carried out by incubating a fixed concentration of apo‐PROSC with increasing concentrations of total *E. coli* RNA, were performed to evaluate the RNA binding capability of this protein in vitro. In addition to total RNA from *E. coli* (Figure 8b), total RNA extracted from human cells (from MDA‐MB‐231, a human breast adenocarcinoma cell line, or from the near‐haploid human cell line HAP1) was used in these analyses (Figure 8c). In all cases, the electrophoretic mobility of apo‐PROSC changed by increasing the concentration of total RNA, but a well‐defined, high molecular weight band, corresponding to a protein–RNA complex, was clearly visible only when human RNA was used (Figure 8c). A greater affinity of apo‐PROSC for RNA extracted from human cells is evident from these analyses. Interestingly, REMSA experiments with apo‐YggS carried out with *E. coli* and human total RNA showed a greater affinity of this protein for the prokaryotic RNA (Figure S6). REMSA analyses were also carried out with both the apo‐ and holo‐forms of PROSC by using biotinylated *E. coli* tRNA, resulting in the formation of multiple protein–RNA complexes (Figure 8d). When tRNA from either *E. coli* or yeast was used in DSF measurements, the thermal stability of apo‐PROSC increased (Figure 8e), suggesting binding of both prokaryotic and eukaryotic RNA to the protein.

### 2.4 Different forms of PLP‐BP are used as baits to capture RNA contained in cell extracts

**FIGURE 9:**
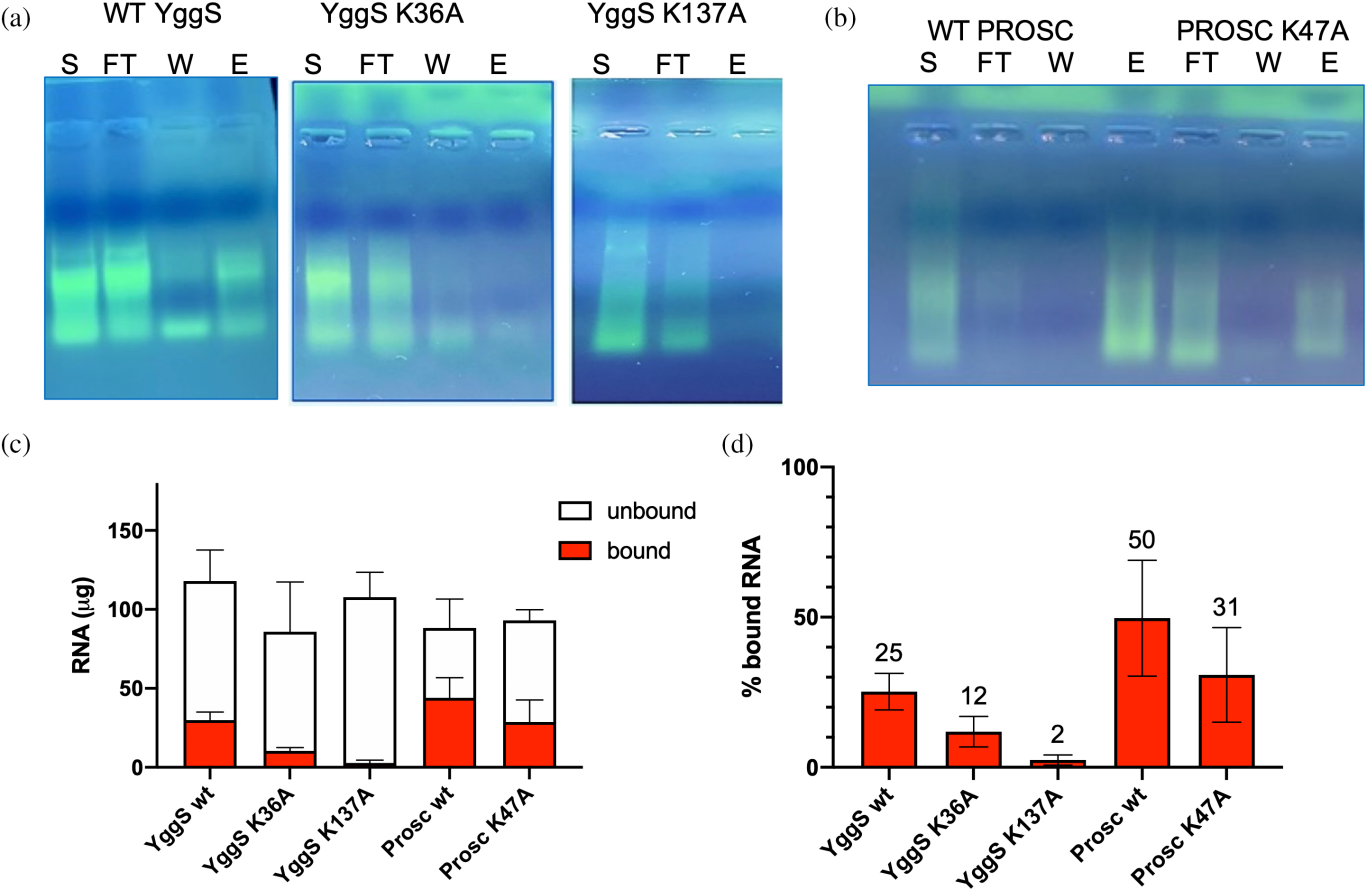
Analysis of RNA capture experiments carried out with WT and variant forms of YggS (a) and PROSC (b) by agarose gel electrophoresis and GelRed staining. As described in the text, whole cell extracts (lanes marked with S in the figure) were loaded onto Ni‐NTA columns on which the proteins had been immobilized, and the unbound material was collected in the flow‐through (FT lanes). This step was followed by a washing step (W lanes), which removed all the residual unbound material, and then by an elution step (E). The RNA collected in the flow‐through, washing and elution steps was separately purified and quantified. Panel c shows the results of the quantification expressed as quantity of RNA that did not bind to the immobilized protein (unbound, collected in the flow‐through and washing steps) and that was retained by the protein (bound, collected in the elution step). Figure d shows the percentage of RNA that was retained by the proteins (% bound RNA) with respect to total RNA (bound + unbound).

The ability of different PLP‐BP forms, immobilized on a Ni‐NTA chromatographic support, to capture RNA contained in cell extracts was assessed. Thirty milligrams of each different PLP‐BP form (RNA free) were loaded onto a 1 mL Ni‐NTA column, previously equilibrated with 20 mM potassium phosphate buffer at pH 7.5 containing 150 mM NaCl, which was subsequently washed with the same buffer. All the protein remained bound to the chromatography support, as checked by measuring the absorbance at 280 nm of the buffer that came out of the column. An equal amount of cell extract (obtained from either BW25113 *E. coli* cells grown to exponential phase or confluent human MDA‐MB‐231 cells, depending on whether the PLP‐BP form immobilized on the column was YggS or PROSC) was then applied to the Ni‐NTA column, and the unbound material was collected in the flow‐through. This step was followed by a washing step with the equilibration buffer and then an elution step with 20 mM potassium phosphate at pH 7.5 containing 1M NaCl (see Section 4 for details). The starting material (cell extract), the flow‐through, the unbound material collected after the washing step, and the material eluted from the column were analyzed by agarose gel electrophoresis followed by GelRed staining. In the case of wild type YggS, most nucleic acid was collected in the flow‐through, however, a significant amount was also retained by the column (Figure 9a). Digestion with DNase and RNase I of the retained nucleic acid showed that it was RNA (Figure S7). In contrast, when the K36A and K137A YggS variants were employed, the nucleic acid retained by the column was much less or even barely detectable (Figure 9a). As a negative control, the *E. coli* FolD protein, recombinantly expressed with a histidine tag, was immobilized onto a Ni‐NTA column and the experiment was repeated in the same conditions. In this case, the eluate did not contain any nucleic acid (data not shown). When wild type PROSC was immobilized on a Ni‐NTA column, and a cell extract of MDA‐MB‐231 cells was passed through the column, most nucleic acid in the starting material remained bound to the protein (Figure 9b). In contrast, when the PROSC K47A variant (corresponding to *E. coli* YggS K36A) was immobilized, most nucleic acid was found in the flow‐through and a smaller amount in the eluate (Figure 9b). The RNA collected in the flow‐through and washing step, and the RNA collected in the eluate from three independent experiments performed with each PLP‐BP form was purified (see Section 4 for details) and quantified (Figure 9c). This quantification showed that the percentage of RNA retained by the protein relative to the total RNA is higher with PROSC wild‐type than with YggS wild‐type. Furthermore, with the PLP‐binding lysine variants K36A YggS and K47A PROSC, the amount of retained RNA is about half compared to wild‐type, and it is about a tenth with K137A YggS (Figure 9d).

### 2.5 RNA sequencing of YggS‐captured RNA

**FIGURE 10:**
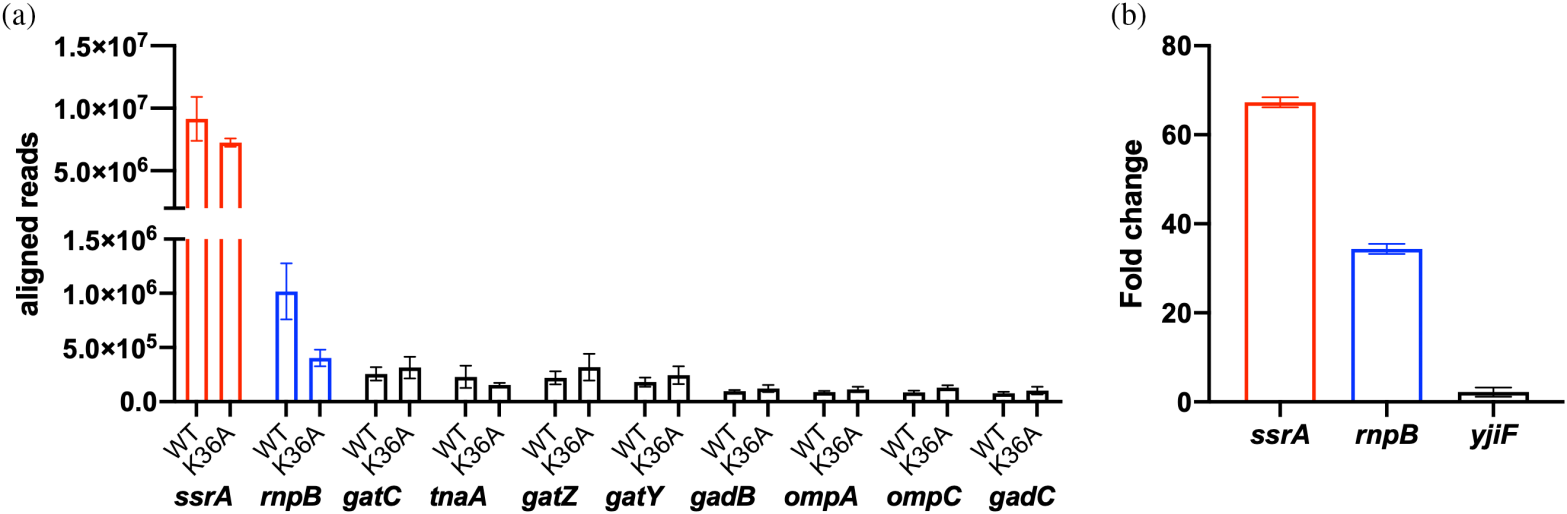
Results of RNA‐seq analyses. (a) Aligned reads returned from the RNA‐seq analysis of the RNA collected and purified from RNA capture experiments that used YggS as bait. Only the first 10 RNA species in descending order of reads are represented in the figure. Reads values are expressed as the average and standard deviation of the results obtained from samples collected in three independent RNA capture experiments. (b) Differential analysis of RNA‐seq data of RNA samples purified from cross‐linked and non‐cross‐linked YggS. The figure shows the fold change value (expressing the quantitative ratio between cross‐linked and non‐cross‐linked RNA species) of the RNA of three genes with an adjusted *p*‐value <0.055, listed by descending order of abundance. Values represent the average and standard deviation from the analysis of three independent RNA‐seq experiments, for both cross‐linked and non‐cross‐linked RNA samples.

The RNA captured, collected and purified from the above experiments by using YggS as bait was analyzed by RNA sequencing (RNA‐seq) after ribosomal RNA depletion (see Section 4 for details), to reveal the identity and measure the relative quantity of RNA molecules. For each protein form (WT, K36A and K137A), the samples obtained from three independent RNA capture experiments were analyzed. As expected, the small amount of RNA captured from the K137A variant proved to be insufficient for the RNA‐seq analysis. Of note, the results obtained with the RNA captured by YggS WT and K36A showed that, in both cases, the transfer‐messenger RNA (tmRNA) encoded by the *ssrA* gene (gene ID BW25113_2621 in Table S1) and the M1 RNA component of ribonuclease P encoded by the *rnpB* gene (gene ID BW25113_3123 in Table S1) were present in much larger amounts than all other detected RNA molecules. Figure 10a shows the number of reads aligning to *ssrA* and *rnpB*, followed by the next eight genes in descending order of abundance. We did not find a significant difference in the abundance of these 10 RNAs between YggS WT and K36A samples, with the exception of the second more abundant RNA (Figure 10a and Table S1). To validate these findings, RT‐qPCR was conducted to measure the relative levels of *SsrA* and *RnpB* RNAs in both bound and unbound fractions. These levels were then compared to those in total RNA extracted from the same samples used to prepare the cell extract applied to the Ni‐NTA column. Results showed that *SsrA* and *RnpB* RNAs were significantly enriched in the bound fraction, while other RNAs, such as *YggS*, *AceB*, and *MnmE*, were not (Figure S8).

### 2.6 RNA‐seq analyses of RNA co‐purified with overexpressed YggS

Given the results obtained from the analysis of RNA captured using YggS as bait, we thought it appropriate to perform a further analysis of the RNA co‐purified with overexpressed YggS from BL21(DE3) containing pET28*yggS* plasmid (Figure 4a). In this analysis, we considered only WT YggS, as the K36A variant yielded very similar results in the RNA‐seq analyses, as described in the previous paragraph. RNA samples isolated from YggS obtained from three separate cultures treated with formaldehyde and three cultures not treated with the cross‐linking agent were subjected to RNA‐seq (see Section 4 for details). Differential analysis of data yielded only three significant differences in gene abundance (adjusted *p*‐value <0.055). The highest fold change value was for *ssrA* (logFC 6.07), followed by *rnpB* and *yjiF* (logFC 5.10 and 1.14, respectively) (Figure 10b and Table S2). These results, together with those previously described in Section 2.5, strongly suggest that the high levels of *SsrA* and *RnpB* RNAs are the result of binding to YggS.

### 2.7 Validation of *ssrA* and *rnpB* as YggS interactors

**FIGURE 11:**
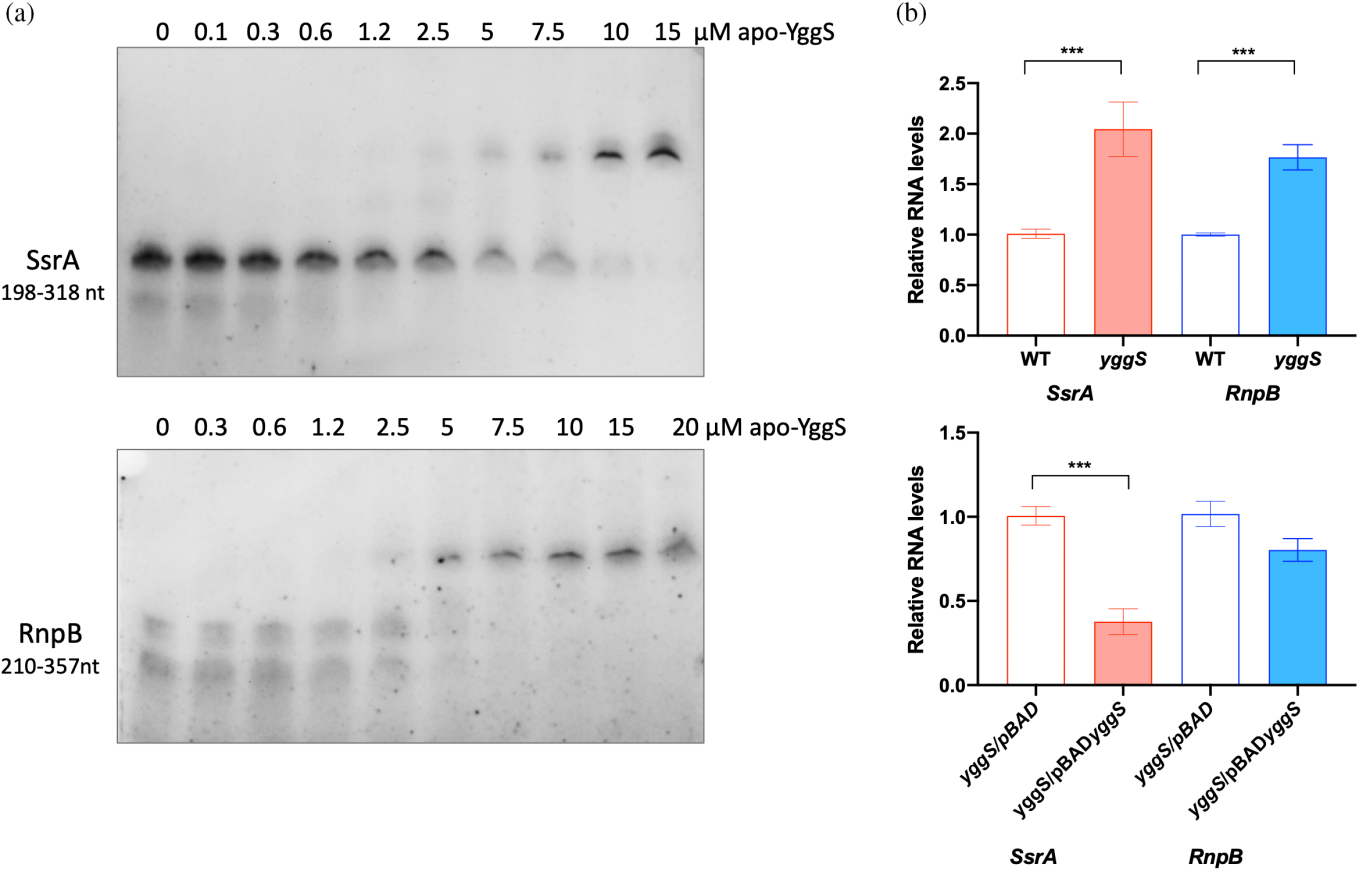
(a) REMSA analysis of YggS binding to *SsrA* and *RnpB* RNAs. REMSA analyses carried out with (20 ng) *SsrA* (upper panel) and *RnpB* (lower panel) RNAs obtained by in vitro transcription (see Section 4.10) incubated with increasing amounts of purified WT apo‐YggS. Naked RNA and YggS–RNA complexes were detected by staining with SyBr Green. (b) Effect of YggS on *SsrA* and *RnpB* RNA levels. The levels of *SsrA* and *RnpB* RNAs in *yggS* deletion strains referred to the wild‐type strain (upper panel) or in *yggS* deletion strain containing pBAD::*yggS* plasmid versus *yggS* deletion strain containing empty pBAD24 plasmid were represented. All bacteria strains were grown in M9 minimal medium to exponential phase. Experimental values are reported as the mean ± SEM. Statistical significance was determined using Student's *t*‐test. *p* values are <0.0005 (***).

By examining closely the RNA‐Seq reads mapped to the *ssrA* and *rnpB* genes using a genome browser (Integrative Genomics Viewer, IGV) (Thorvaldsdottir et al., 2013), we identified the regions that are most represented in the mapped RNA reads. Concerning *ssrA*, this region is sharply defined, and encompasses positions 198 and 318 within the 363‐nucleotide sequence, whereas the 210–357 region is the most represented in the 377‐nucleotide sequence of *rnpB* (Figure S9). Both RNA fragments were produced by in vitro transcription and used in REMSA analyses carried out in the presence of purified WT apo‐YggS. Compared to tRNA (Figure 6a), YggS showed a higher affinity for these in vitro‐produced RNAs (Figure 11a). The apparent *K*_d_ estimated from the densitometric measurements of the faster bands (free RNA) were of 2.8 ± 0.2 μM for binding to *SsrA* and 3.4 ± 0.2 μM for binding to *RnpB*. Additionally, PLP negatively affected YggS binding to these RNAs (data not shown), as observed for the tRNA binding (Figure 6a).

Moreover, the levels of *SsrA* and *RnpB* RNAs were measured by RT‐qPCR in WT and *yggS* deletion *E. coli* strains grown in both M9‐glucose 0.4% minimal medium and LB rich medium. The results show that both RNAs are significantly more abundant in the deletion strain with respect to the WT strain (Figure 11b, upper panel). For comparison, RNAs of a series of genes that are involved in PLP metabolism, such as *pdxB*, *pdxJ*, *pdxK*, *pdxI* and *pdxH*, are instead present at the same levels in the two *E. coli* strains (Figure S10). Complementation of the deletion mutant by expression of YggS in trans restores *SsrA* and *RnpB* RNA levels to the values observed in the wild‐type strain (Figure 11b, lower panel).

### 2.8 Prediction of RNA‐binding residues and evolutionary conservation analysis of PLP‐BP from different sources

**FIGURE 12:**
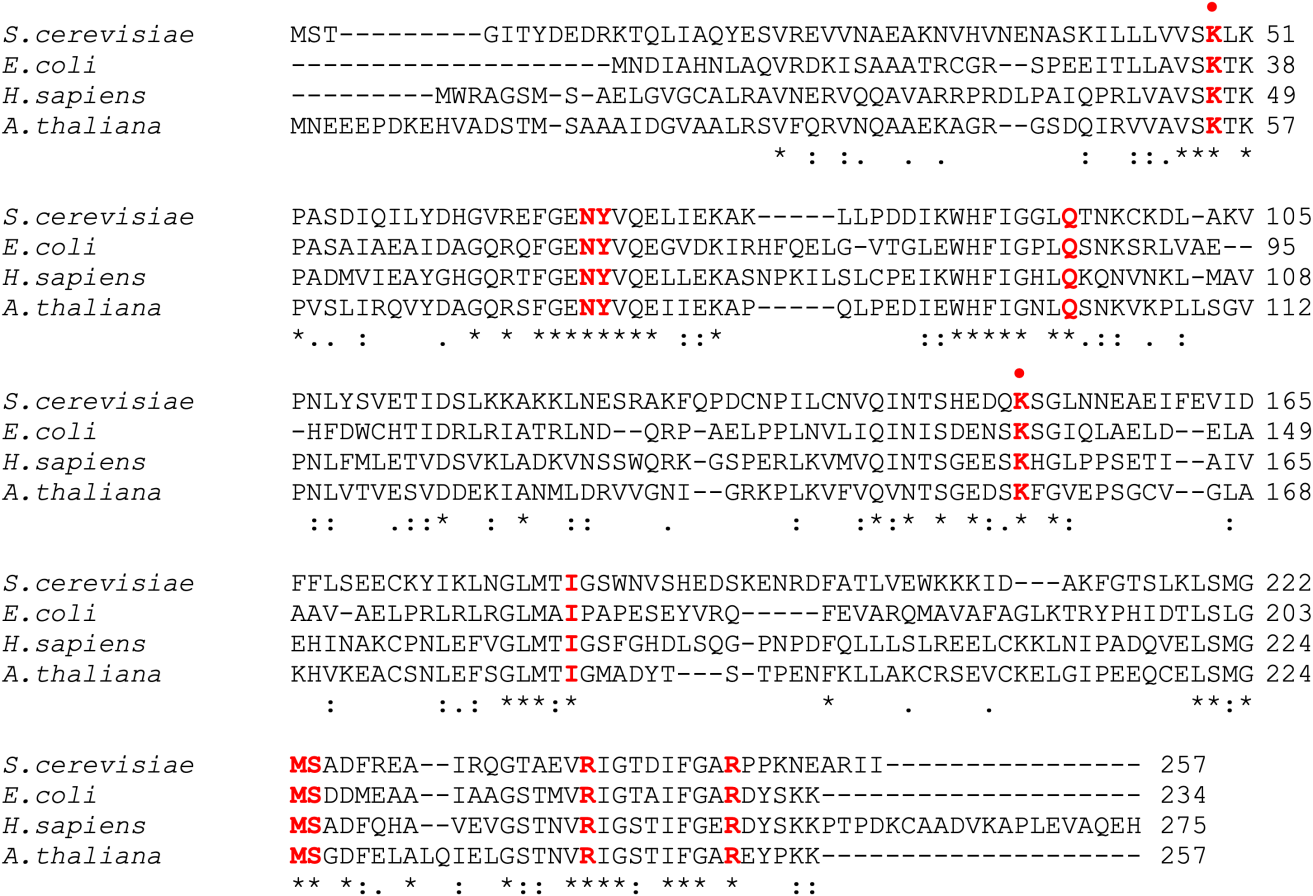
Multiple sequence alignment of PLP‐BPs from *Saccharomyces cerevisiae*, *Escherichia coli* (YggS), *Homo sapiens* (PROSC) and *Arabidopsis thaliana*. The 10 common residues in the four proteins that are predicted to bind RNA and at the same time show a high evolutionary conservation in the ConSurf analysis are shown in bold red. K36 and K137 residues are indicated by a red dot. The multiple alignment was obtained using the EMBL‐EBI Clustal Omega multiple sequence alignment program (https://www.ebi.ac.uk/jdispatcher/msa/clustalo).

**FIGURE 13:**
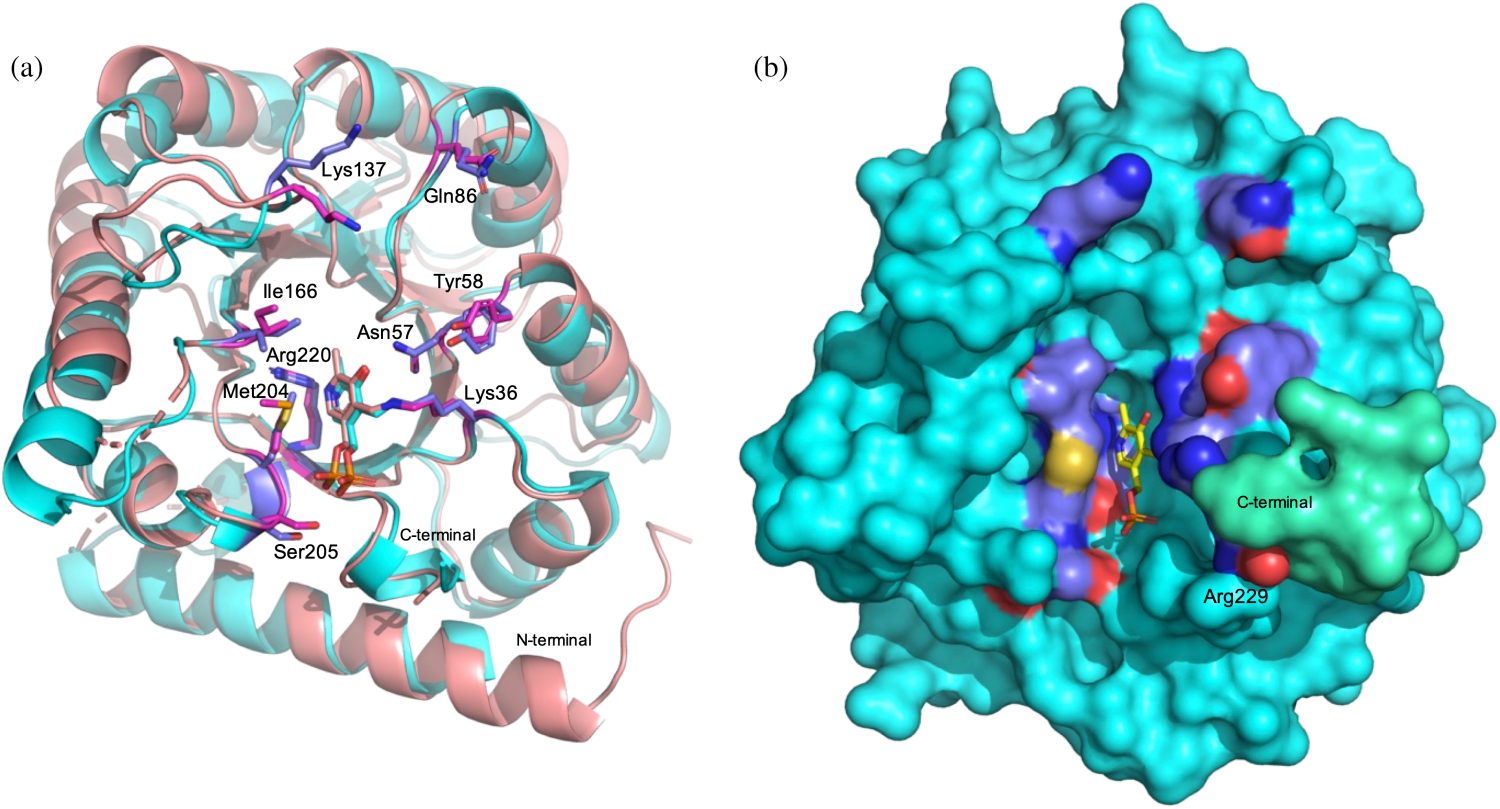
Location of the 10 common predicted RNA‐binding residues on the crystal structures of YggS and *S. cerevisiae* PLP‐BP. (a) Superimposition of YggS (PDB: 1W8G; in cyan) and PLP‐BP from *S. cerevisiae* (PDB: 1CT5; in salmon) in which the predicted RNA‐binding residues are shown as sticks (with carbon atoms in slate for YggS and in magenta for *S. cerevisiae* PLP‐BP; CPK coloring is applied to all other atoms) and numbered according to YggS sequence. PLP is represented as sticks. Arg229 in YggS and the corresponding Arg250 in *S. cerevisiae* PLP‐BP are not shown in the structures since the C‐terminal end of both proteins is absent in the electron density maps. (b) Protein surface representation of the YggS crystal structure shown in panel a, with the same color code and orientation. Only PLP is shown as sticks with yellow carbon atoms. In the figure, the C‐terminal end of the protein (with sequence RDYSKK, from residue 229 to 334), which is absent in the crystal structure, was modeled using AlphaFold and is represented in greencyan. Arg229 is shown with slate carbon atoms and is labeled in the figure.

GPSite (https://bio-web1.nscc-gz.cn/app/GPSite) (Yuan et al., 2024), a recently developed geometry‐aware multi‐task network for simultaneously predicting binding residues of biologically relevant ligands (DNA, RNA, peptide, protein, ATP, HEM and metal ions), was used to identify possible RNA‐binding residues in PLP‐BP from different and evolutionary distant sources: *E. coli*, *Saccharomyces cerevisiae*, *Homo sapiens* and *A. thaliana* (see Section 4 for details). Moreover, the ConSurf web‐server (https://consurf.tau.ac.il/consurf_index.php) (Yariv et al., 2023) was used to identify conserved regions of structural and functional importance, in each of the four PLP‐BPs analyzed, based on multiple sequence alignments of homologous proteins in phylogenetically related organisms (see Section 4 for details). In the GPSite analysis of the examined proteins, RNA and DNA showed the highest binding probabilities, which ranged from 0.910 to 0.934 and from 0.930 to 0.951 on a 0–1 scale, for RNA and DNA respectively. Protein binding probabilities were also relatively high, ranging from 0.818 to 0.925. In contrast, other ligands exhibited much lower binding probabilities, ranging from 0.056 to 0.547. When examining the residue‐level annotation, we focused on RNA binding and considered the residues scoring above 70 in *E. coli* YggS (Table 1). The combination of GPSite and ConSurf analyses led to the identification of 10 common residues among the four proteins that are predicted to bind RNA and at the same time show a high evolutionary conservation according to ConSurf scale (Figure S11). Referring to YggS amino acid numbering, these residues are K36, N57, Y58, Q86, K137, I166, M204, S205, R220 and R229 (Table 1 and multiple alignment in Figure 12). Since both the crystal structures of YggS and *S. cerevisiae* PLP‐BP, which are very similar, are available (PDB IDs: 1W8G and 1CT5, respectively), it can be observed that all 10 of the identified residues are located on the surface of the proteins, at the edge of the PLP binding site, and can therefore play a functional role such as interacting with a ligand (Figure 13). Differently, the crystal structures of human and plant PLP‐BPs are not available, but their three‐dimensional structure models can be obtained from the AlphaFold Protein Structure Database (https://alphafold.ebi.ac.uk/; O94903 PLPHP_HUMAN and Q944L8_ARATH, respectively). These 3D models overlap well with the 3D structures of YggS and yeast PLP‐BP. However, the N‐terminal and C‐terminal regions are more extended in the eukaryotic proteins than in YggS (multiple alignment in Figures 12 and S12). In particular, the N‐terminal end of the plant protein (as also observed in Farkas and Fitzpatrick (2024)) and the C‐terminal end of the human protein are about 20 residues longer than those in YggS. Interestingly, these regions in the 3D AlphaFold models have per‐residue model confidence scores (pLDDT) that are very low (<50), suggesting that they may be unstructured. As a matter of fact, the AIUPred web interface (https://iupred.elte.hu/) (Erdos & Dosztanyi, 2024), which identifies disordered protein regions, predicts a high disorder tendency of the first 15 N‐terminal residues for *A. thaliana* PLP‐BP (Figure S13), with scores from 0.61 to 0.84 (on a 0–1 scale), where most residues of the protein have scores around 0.2. AIUPred can also identify (via the ANCHOR2 tool) protein regions that may or may not adopt a stable structure depending on ligand binding. This analysis assigns the highest scores to the N‐terminal region compared to the rest of the polypeptide chain (Figure S13). Consistently, the AIUPred web interface predicts a higher disorder tendency for the C‐terminal end of human PLP‐BP compared to the rest of the polypeptide chain, albeit less pronounced than in the homologous plant protein (Figure S13). Notably, for all four PLP‐BPs examined, the polypeptide loop containing YggS K137 is located in a region of the disorder tendency graph where a relative peak is observed (loops 130–141 in YggS; 143–156 in yeast; 142–157 in *H. sapiens*; 148–160 in *A. thaliana*).

**TABLE 1:** RNA binding propensities of residues of PLP‐BP from different sources, as predicted by the GPSite server (https://bio‐web1.nscc‐gz.cn/app/GPSite) (Yuan et al., 2024).

*E. coli*		*S. cerevisiae*		*H. sapiens*		*A. thaliana*	
Residue	Score	Residue	Score	Residue	Score	Residue	Score
**K36**	**93**	**K49**	**94**	**K47**	**95**	**K55**	**93**
**N57**	**90**	**N70**	**93**	**N68**	**92**	**N76**	**92**
**Y58**	**90**	**Y71**	**92**	**Y69**	**93**	**Y77**	**92**
P84	88	G93	82	H96	95	N99	87
**Q86**	**91**	**Q95**	**92**	**Q98**	**95**	**Q101**	**92**
S87	74	T96	55	K99	86	S102	65
N88	82	N97	82	Q100	80	N103	80
K89	81	K98	83	N101	63	K104	85
**K137**	**88**	**K151**	**85**	**K153**	**82**	**K156**	**86**
S138	71	S152	63	H154	89	F157	85
**I166**	**96**	**I183**	**96**	**I183**	**96**	**I186**	**93**
P167	83	G184	78	G184	73	G187	58
A168	77	S185	80	S185	74	M188	54
P169	82	W186	90	F186	93	A189	77
**M204**	**96**	**M223**	**95**	**M225**	**96**	**M225**	**94**
**S205**	**84**	**S224**	**78**	**S226**	**87**	**S226**	**93**
**R220**	**87**	**R239**	**83**	**R241**	**80**	**R243**	**81**
T223	80	T242	82	S244	85	S246	76
**R229**	**93**	**R248**	**92**	**R250**	**90**	**R252**	**92**

*Note*: The table has been compiled on the basis of residues scoring >70 in *E. coli* YggS. In the columns, the residues are ordered according to *E. coli* YggS numbering. Residues in the same row occupy the same position in the multiple alignment shown in Figure 11. The most conserved residues indicated by the ConSurf analysis, which are invariant in the multiple alignment and also show the highest scores (≥78), are 10 and are shown in bold.

## 3 DISCUSSION

The ability of YggS to bind RNA is evident from our experiments. However, a key question concerns the specificity of this binding. In other words: is the binding to RNA accidental and nonspecific, merely depending on the presence of positively charged residues on the surface of the protein, or is it the result of a genuine, specific interaction between the protein and RNA? Answering this question is crucial, as it implies whether the RNA binding to YggS has a specific function or not. An affirmative answer would represent a significant step towards understanding the elusive role of this ubiquitous protein, and could open up new horizons in the field of vitamin B_6_ metabolism.

Formaldehyde cross‐linking (Figure 1) may have induced binding of RNA that was not directly and tightly bound to the protein. However, binding of abundant RNA to the protein was also observed by UV cross‐linking (Figure 3), and it is known that this technique only captures RNA directly bound to the protein at the time of irradiation (Urdaneta & Beckmann, 2020), which occurs during a very brief pulse. The fact that human PROSC (Figure 8) and plant PLP‐BP (data not shown) recombinantly expressed in *E. coli* also co‐purify with bound RNA argues in favor of evolutionarily conserved binding. In addition, our REMSA analyses with YggS (Figure S6) and PROSC (Figure 8b,c) show that these proteins bind RNA extracted from cells of their own source (*E. coli* and human cells, respectively) with the highest affinity. It is very notable that the apo‐forms of both YggS (Figures 5 and 6) and PROSC (Figure 8d) bind RNA with much greater affinity than the holo‐forms. This observation inevitably suggests a possible role for PLP as an effector molecule regulating the binding of RNA to these PLP‐BPs. Another important point is the observed lower affinity of the K36A and K137A variants for RNA (Figures 4, 5, 6, 7). The K36A variant, which is unable to bind PLP in the canonical site, may exhibit reduced RNA binding because, as discussed in Section 2.2, it adopts a holo‐like conformation even in the absence of PLP. In contrast, the K137A variant retains the same PLP binding properties as the WT protein. However, in vivo experiments showed that the K137A variant is unable to complement the phenotype of an *E. coli yggS* deletion strain (Tramonti et al., 2022). Taken together, these observations suggest that Lys137 is directly involved in RNA recognition.

RNA binding is also evident from RNA capture experiments (Figure 9). In these experiments, the immobilized WT forms of YggS and PROSC were shown to bind RNA with high affinity from crude *E. coli* and human cell extracts, respectively. Again, the YggS and PROSC variants showed a much lower RNA affinity than their WT counterparts. In particular, the K137A form of YggS bound only a very small amount of RNA with respect to the WT form. It should be noted that the proteins immobilized on the chromatographic support certainly contained a significant portion of the apo‐form, given their natural tendency to lose PLP during purification (Tramonti et al., 2022).

The results of RNA‐seq analyses of the material isolated in the RNA capture experiments and co‐purified with YggS overexpressed in *E. coli* cells treated or not with formaldehyde (see paragraph 2.6) showed that two RNA species, namely the *SsrA* tmRNA and the *RnpB* RNA, are much more abundant than all the others (Figure 10). *SsrA* or transfer‐messenger RNA (tmRNA) is a specialized RNA molecule that, together with the small protein B (SmpB), plays a crucial role in trans‐translation, the primary mechanism for rescuing stalled ribosomes in bacteria. The tmRNA–SmpB complex mimics the structure of a tRNA molecule, allowing tmRNA to be aminoacylated with alanine, and engages with the ribosome. The mRNA‐like domain of *SsrA* contains an internal open reading frame, that encodes a tag, which is added to the incomplete, stalled peptide. This tag signals proteases for targeted degradation of the faulty peptide (Guyomar et al., 2021). *RnpB*, or M1 RNA, is the catalytic subunit of the RNase P ribonucleoprotein, a tRNA processing enzyme responsible for removing 5′ leaders from precursor tRNAs to produce mature 5′ termini. RNase P is an essential ribonucleoprotein found in all kingdoms of life. In bacteria, *RnpB* functions as a ribozyme, exhibiting catalytic activity even in the absence of its protein component, SmtB (Frank & Pace, 1998). As mentioned in the RNA‐seq results section, the region of *SsrA* which is most represented in the mapped RNA reads is that comprising positions 198 and 318 of the 363‐nucleotide sequence. This is the RNA region that is supposed to be recognized by YggS. It is interesting to note that in the cryo‐EM structure of the trans‐translational complex on the stalled *E. coli* ribosome (Guyomar et al., 2021), this RNA region is the only one without bound proteins (Figure S14). Of great importance for the possible function of YggS is the fact that both RNA species bound by this protein play a crucial role in protein translation. In particular, *RnpB* is an essential RNA in *E. coli*. It is important to note that after ribosomal RNA, *SsrA* and *RnpB* are considered among the most abundant RNA species in *E. coli* (Keiler, 2008). Therefore, the result of our RNA‐Seq analyses, which identified these RNAs as the most represented in RNA captured by YggS or cross‐linked to YggS, could be due to this. However, the hypothesis that YggS interacts with these two RNA species seems to be supported by the observation that the absence of YggS in the *E. coli yggS* deletion strain affects the levels of *SsrA* and *RnpB*, as shown by our RT‐qPCR results obtained with respect to WT (Figure 11b).

It has been shown that deletion of the *ssrA* gene results in increased sensitivity to streptomycin (Abo et al., 2002). Streptomycin functions as a protein synthesis inhibitor by binding to the 30S ribosomal subunit, causing miscoding of termination codons. Abo et al. (2002) have shown that even at sublethal concentrations, streptomycin increases the amount of proteins tagged by *SsrA* tmRNA, highlighting the role of trans‐translation in correcting translational errors induced by miscoding antibiotics. When we tested whether the *yggS* deletion strain had an increased sensitivity to some of the most commonly used antibacterial compounds (rifampin, erythromycin, vancomycin, tetracycline, streptomycin, chloramphenicol), we found a significantly increased sensitivity to streptomycin (Figure S15). Therefore, this observation is also an indication of a link between the role of *SsrA* and the function of YggS. It should be noted that *yggS* is not an essential gene in *E. coli*, either in rich or minimal media. However, it is possible that YggS may become physiologically important under growth and nutrient availability conditions different from those tested so far, including stress conditions.

Prediction of RNA‐binding residues and the evolutionary conservation analyses strongly support the idea of RNA specifically binding to PLP‐BPs and playing a crucial functional role across evolutionary distant sources. The residues predicted to bind RNA (Table 1), which are also among the most conserved in PLP‐BPs from different sources (Figure S11), are aligned at the edges of the protein PLP‐binding site (Figure 13). Among them is Lys137, placed in a polypeptide loop that is predicted to be flexible or unstructured (loops 130–141 in YggS, 143–156 in yeast, 142–157 in *H. sapiens*; 148–160 in *A. thaliana*; Figure S13). It is therefore very tempting to think that these residues may define the binding site of RNA and that, depending on whether PLP is bound or not at the PLP‐binding site, this site may change conformation, thereby modulating the binding affinity for RNA. The observation that the analyzed PLP‐BPs have a markedly different extent of their C‐ and N‐terminal ends (Figures 12 and S12) may be relevant to the specific binding of RNA. In fact, these regions, which in eukaryotic proteins have a high probability of being disordered, have a propension to become structured following the interaction with a specific RNA molecule (Figure S13). Therefore, this feature, in front of a common (or very similar) RNA binding site located at the entrance of the PLP‐binding site, could confer binding specificity to PLP‐BPs from evolutionarily distant organisms. It follows that although RNA binding is highly conserved in PLP‐BPs, the specificity for RNA and therefore the regulatory function performed by each protein could be different. Consider for example that mutations in the PROSC gene cause a severe form of epilepsy in humans (Darin et al., 2016; Johnstone et al., 2019; Plecko et al., 2017; Shiraku et al., 2018), whereas the knockout of *yggS* in *E. coli* have no apparent effect on growth capabilities in either rich or minimal liquid growth medium (Babor et al., 2023).

We are certainly a long way from understanding the actual function of YggS and other PLP‐BPs. However, we suppose that our observations represent a clear and novel indication that the protein interacts with RNA and that this interaction, conserved between evolutionarily distant species, most likely has a physiological function. Concerning YggS, the hypothetical interaction of this protein with RNA species that play an important role in ribosomal translation, and the observation that this interaction is stronger in the absence of PLP, suggest that YggS may be part of a regulatory mechanism of translation linked to amino acid levels, which are in turn strictly connected to PLP metabolism. Further efforts will be devoted to identify specific interactors of other PLP‐BPs, and unravel the physiological role of such interactions.

## 4 METHODS

### 4.1 Expression and purification of WT and variant YggS and PROSC forms

The coding sequence of the human PROSC (isoform 2; NP_009129.1) has been synthesized from GenScript (Leiden, Netherlands), optimized for expression in *E. coli* cells and inserted into a pET28a(+) vector at the cloning sites NdeI and XhoI. This new construct, named pET28‐PROSC, was used to transform *E. coli* BL21(DE3) competent cells for protein expression. The K47A PROSC variant was produced by site‐directed mutagenesis carried out using a standard protocol described in the Quick‐Change kit from Stratagene (La Jolla, CA) and two complementary oligonucleotide primers, synthesized by Metabion International AG (Steinkirchen, Germany), with sequence 5′‐GTGGCGGTTAGCGCAACCAAACCGGC‐3′ and 5′‐GCCGGTTTGGTTGCGCTAACCGCCAC‐3′. The purification of WT and K47A PROSC was carried out using the same protocol of all YggS forms (Tramonti et al., 2022). Briefly, an overnight culture (5 mL) of *E. coli* BL21(DE3) cells transformed with appropriate plasmids was used to inoculate 1 L of LB medium. Bacteria were allowed to grow at 37°C until their OD600 reached to ~0.6, then the growing temperature was lowered to 28°C and the expression of either YggS or PROSC induced with 0.2 mM isopropyl thio‐β‐D‐galactoside (IPTG). Bacteria were harvested after 18 h and suspended in 50 mL of 20 mM KPi pH 7.5 buffer, containing 150 mM NaCl and 2.5 mM 2‐mercaptoethanol (buffer A) 1 mM PMSF and 2 mg/mL lysozyme. Cell lysis was carried out with three freezing/defrosting cycles and by sonication on ice (3‐min in short 20‐s pulses with 20‐s intervals). Lysate was centrifuged at 12,000*g* for 30 min and the supernatant was loaded onto a 1 mL Ni‐NTA HisTrap (Cytiva) column, previously equilibrated with buffer A. The column was washed with 5 mL of buffer A and eluted with the same buffer and a linear 0–300 mM imidazole gradient (the buffer containing imidazole was adjusted to pH 7.5 using HCl). Fractions containing the proteins were collected and dialyzed against buffer A. The recombinant PROSC (molecular weight 31,167 Da) presents an N‐terminal 6x His tag.

The wild type and variant apo‐forms of YggS and PROSC were prepared using L‐cysteine as described in Tramonti et al. (2022). All proteins were kept in buffer A.

When the YggS and PROSC forms were used in REMSA, nucleic acids were removed by adding RNase I to the lysate and by washing the Ni‐NTA column with 10 mL of 20 mM KPi pH 7.5 buffer, containing 1 M NaCl and 2.5 mM 2‐mercaptoethanol.

FolD, *e*SHMT, L‐TA were expressed and purified as previously described (Contestabile et al., 2001; Fu et al., 2001). *A. thaliana* PLP‐BP was expressed and purified as described for YggS.

### 4.2 In vivo cross‐linking experiments

The *E. coli* BL21(DE3) containing the pET28*yggS* plasmid was grown at 37°C and after 4 h induced with IPTG (0.2 mM) and then kept at 28°C for 16 h. When formaldehyde was used as cross‐linking agent, this was added to the cultures to have 1% final concentration. The cultures were incubated at room temperature for 20 min and then cross‐linking was quenched by adding 0.5 M glycine.

UV‐cross‐linking was obtained by irradiating the bacterial cells resuspended in cold PBS and poured into 15 cm Petri dishes. Cells were exposed to 3 × 333 mJ/cm^2^ UV radiation (*λ* = 254 nm) in a Spectrolinker XL‐1000 UV cross‐linker (Spectronics Corporation; New York, USA).

### 4.3 Western blot analysis

Samples were run on 12% SDS‐PAGE and directly electroblotted onto nitrocellulose membrane (Bio‐Rad, Hercules, CA, USA). YggS was detected with monoclonal anti‐polyhistidines antibodies (Sigma) using the TMB ready‐to‐use substrate (Serva, Heidelberg, Germany).

### 4.4 RNA purification

Total RNA was isolated from BW25113 *E. coli* or human MDA‐MB‐231 and HAP1 cell cultures using the NucleoSpin RNA kit from Macherey and Nagel (Hoerdt, France). Low molecular weight RNA was purified from BW25113 *E. coli* cells using PureLink miRNA Isolation kit (Invitrogen, Waltham, MA, USA). RNA bound to proteins was purified using a total RNA purification kit from Norgen Biotek (Thorold, Ontario, Canada). RNA quality and concentration were evaluated by measuring the absorbance at 260 nm and the 260/280 nm ratio in 0.1M NaOH, and by electrophoresis on 1.2% agarose gel or 10% acrylamide gel in the presence of 6.5M urea gels. Yeast tRNA was purchased from Invitrogen.

### 4.5 RNA electrophoretic mobility shift assays

RNA mobility shift assays were conducted using purified apo‐YggS and PROSC WT and variant forms. When the holo‐forms were used, 5‐fold excess PLP with respect to protein was added so as to ensure complete saturation. Proteins (1 μg) were incubated at 22°C for 5 min in 10 μL binding buffer (20 mM HEPES, pH 8.0, containing 50 mM KCl, 5 mM MgCl_2_, 1 mM dithiothreitol (DTT), 0.05% (v/v) NP‐40, 30 μg/mL BSA and 5% (v/v) glycerol) with increasing concentrations of RNA. Samples were loaded onto 5% non‐denaturing polyacrylamide gels in 0.5× TBE buffer (45 mM Tris‐borate, 1 mM EDTA). Gels were run at room temperature in 0.5× TBE buffer and then incubated with Quick Coomassie Stain (Serva) for 15 min.

For the REMSA carried out with fixed amount of RNA and increasing concentrations of proteins, the RNA was labeled at the 3′ end with a molecule of cytidine‐5′‐phosphate‐3′‐(6‐aminohexyl)phosphate labeled with biotin (pCp‐biotin) using the Pierce™ RNA 3′ End Biotinylation Kit (Thermo Scientific, Waltham, MA, USA) and purified according to manufacturer instructions.

All the components were incubated at RT for 30 min in 10 μL of binding buffer (10 mM HEPES pH 7.3, 10 mM KCl, 1 mM MgCl_2_, 1 mM DTT, 5% glycerol). The reaction mixtures were separated onto 5% nondenaturing polyacrylamide gels in 0.5× TBE buffer. To detect biotin‐labeled RNA, gels were transferred onto Biodyne positive charged nylon membranes (0.4 μm; Thermo Scientific) in 0.5× TBE, the RNA was then cross‐linked to the membrane using an UV cross‐linker equipped with 312 nm bulbs (Spectroline, Westbury, New York). The biotin moiety was detected by chemiluminescence using the Chemiluminescent Nucleic Acid Detection Module (Thermo Scientific) in a Chemidoc MP Imaging System (Bio‐Rad). Densitometric measurements of the bands corresponding to free RNA molecules, obtained with the ImageLab software (Bio‐Rad), were transformed into percentages, the amount of bound RNA (% bound) was then calculated by subtracting the percentage of free RNA from the total, and the result was plotted as a function of protein concentration. The apparent dissociation constants were estimated by fitting the data to a hyperbolic equation.

### 4.6 Differential scanning fluorimetry assays

DSF assays were performed on a Real Time PCR Instrument (CFX Connect Real Time PCR system, Bio‐Rad). In a typical experiment, 2 μM apo‐YggS and PROSC forms in buffer A, and Sypro Orange (5×, Thermo Scientific) were mixed with RNA (total volume of 25 μL) in a 96‐well PCR plate. Fluorescence was measured from 25 to 95°C in 0.4°C/30 s steps (excitation 450–490 nm; detection 560–580 nm). All samples were run in triplicate. Denaturation profiles were analyzed according to the Boltzman equation as described in Tramonti et al. (2022).

### 4.7 Gel filtration analyses

Gel filtration of cross‐linked and non‐cross‐linked YggS was performed on a Superdex 200 10/300 GL column (Cytiva, Marlborough, MA, USA) at room temperature and at a flow rate of 0.5 mL/min in buffer A. Elution profiles were obtained by following the absorbance at 280 nm. Calibration was performed with aldolase (158 kDa), conalbumin (75 kDa), ovalbumin (44 kDa), carbonic anhydrase (29 kDa) and RNase A (17.7 kDa).

### 4.8 RNA sequencing

Concerning samples obtained in capture experiments, the RNA collected in the NaCl eluate from three independent experiments performed with WT and K36A YggS forms was treated with DNase I (RNase free) and purified (using the RNA clean‐up and concentration kit by Norgen). All RNA samples were subjected to ribosomal RNA depletion. Library construction and IlluminaNovaSeq 6000 system‐based sequencing were performed by Procomcure Biotech GmbH (Thalgau, Austria). Concerning RNA samples isolated from overexpressed WT YggS, the protein was purified from three separate cultures treated with formaldehyde and three cultures that were not treated with the cross‐linking agent. RNA was purified from protein samples using the NucleoSpin RNA kit from Macherey and Nagel and subjected to ribosomal RNA depletion. Library construction and IlluminaNovaSeq 6000 system‐based sequencing were performed by IGATech (Udine, Italy).

### 4.9 Preprocessing and analysis of RNA sequencing data

Preprocessing of RNA‐seq data was performed in Galaxy (Galaxy version 24.1.dev0) (Galaxy, 2024). Specifically, we used FastQC (version 0.12.1) (https://www.bioinformatics.babraham.ac.uk/projects/fastqc/) to assess read quality before and after trimming, Trimmomatic (version 0.39) (Bolger et al., 2014) to remove low‐quality reads and adapter traces, HISAT2 (version 2.2.1) (Kim et al., 2015) for read mapping, and FeatureCounts (Subread version 2.0.3; Samtools version 1.16.1) (Liao et al., 2014) for summarizing read counts on annotated gene features. Reads were mapped against the *E. coli* K‐12 substr. BW25113 genome (GenBank assembly ID: GCA_000750555.1) or the *E. coli* BL21(DE3) genome (GenBank assembly ID: GCA_000022665.2), depending on the source of the sequenced RNA. Genome annotations were also taken from GenBank. All listed software were used with default parameters through the Galaxy interface except for HISAT2, in which the “spliced alignments” option was disabled (“disable spliced alignments = true”). Differential expression analysis for the cross‐linking experiments was also performed in Galaxy by using the limma‐voom software (Law et al., 2014; Liu et al., 2015). Mapped reads were visualized using the IGV (Integrative Genomics Viewer, version 2.17.4) software (Thorvaldsdottir et al., 2013).

### 4.10 In vitro transcription

The sequences of *ssrA* (198–318 nt) and *rnpB* (210–357 nt) were amplified by PCR using *E. coli* genomic DNA as template and primers designed to include the T7 promoter sequence upstream of each fragment: for *ssrA* tgtaatacgactcactatagggTCGCGTGGAAGCCCTGC (forward) and CCTCGGTACTACATGCTTAGTC (reverse), whereas for *rnpB* tgtaatacgactcactatagggTGGTAACAGTCCGTGGCAC (forward) and CCGGGTTCTGTCGTGGAC (reverse). PCR products were purified from agarose gel using the PCR Clean‐up Kit (Macherey‐Nagel). The DNA fragments generated by PCR were used as templates to produce the corresponding RNA sequences with Ribomax Large Scale RNA Production System‐T7 Kit (Promega, Madison, WI, USA). The produced RNA was purified using RNA Clean‐Up and Concentration Kit (Norgen Biotek) and quantified by measuring the absorbance at 260 nm in 0.1M NaOH.

### 4.11 Quantitative RT‐PCR analysis

RNA was isolated from three independent cultures (three biological replicates), grown to exponential or stationary phase, using the NucleoSpin RNA kit from Macherey and Nagel. RNA quality and concentration were evaluated by measuring the OD at 260 nm and the 260/280 nm ratio in 0.1M NaOH, and by electrophoresis on 1.2% agarose gels. RT‐qPCR reactions were performed in two steps. Reverse transcription of DNase‐treated RNAs (0.5 μg) was carried out using the OneScript® Plus cDNA Synthesis Kit (ABM) with the random primers provided in the kit. Real Time PCR was performed on a Real Time PCR Instrument (CFX Connect Real Time PCR system, Bio‐Rad) with a two‐step reaction using PowerTrack™ SYBR Green Master mix (Applied Biosystem) and the following oligonucleotides:

*ssrA*: CCTGCCTGGGGTTGAAGC and GTCAGTCTTTACATTCGCTTGCC.

*rnpB*: GTGGCACGGTAAACTCCACC and CAGCAATCGCTCACTGGCTC.

*pdxB*: ATTGGTTTTTCCGCTGCACC and CGCGCTGGACTAACTCATCC.

*pdxJ*: TGACCATATCGCTACGCTGC and CCAGGCAGCAAAAATGTGGC.

*pdxH*: ATGGTGGTCGCTACCGTGG and CTATCACGCGGGCGGCTATG.

*pdxK*: TGCCGTGCCTGCTATCAAAC and AGGATGGTCTTTGCGTAGCG.

*pdxY*: AATGGACTGGCTGCGTGATG and GCACATGAAACTCTGCGACACC.

*ybhA*: GCTTCTCTGGCTGAAACGGC and AAGTTATCGCCGAATGCCACG.

*pdxI*: CCCAGCGGAAGGATCGATTG and CCATCGTGGGCCAAAGCATC.

*glyA*: CCGCAAAGTTAGCCTGGGAG and CATCGAACTGATCGCCTCCG.

*recA*: GATTTCCAGTGCCTGCTCGC and ACTGGATATCGCGCTTGGGG.

*gyrB*: AGAATCGCCTGGTTCTTGCG and TGCTGGAAAACCCAACCGAC.

The relative level of each specific RNA was determined by the Pfaffl method using the *recA* and *gyrB* genes as normalizers. The fold induction resulting from the different pairs of samples was averaged and the *p*‐value was calculated using the Student's *t*‐test.

### 4.12 Prediction of RNA‐binding residues and evolutionary conservation analysis of PLP‐BP from different sources

Concerning the GPSite analysis, the input protein sequences were: for *E. coli* YggS, GenBank CAD6004200.1; for *S. cerevisiae* PLP‐BP, UniProtKB/Swiss‐Prot P38197.1; for human PROSC (pyridoxal phosphate homeostasis protein isoform 2), NCBI Reference Sequence NP_009129.1; for *A. thaliana* PLP‐BP (pyridoxal phosphate homeostasis protein isoform 1, At1g11930/F12F1_20), UniProtKB/Swiss‐Prot Q944L8. GPSite provided the following results, accessible via the specified links: for *E. coli* YggS, https://bio‐web1.nscc‐gz.cn/job/result/e778dd33‐e551‐4026‐a43e‐c00464c47d8e/yggS; for *S. cerevisiae* PLP‐BP, https://bio‐web1.nscc‐gz.cn/job/result/5a3140d7‐61cd‐4345‐8eb2‐639710908d23/sp_P38197; for human PROSC, https://bio‐web1.nscc‐gz.cn/job/result/9dbf9a01‐373f‐4869‐8dbe‐51284c571b15/NP_009129.1_pyridoxal_phosphate_homeostasis_protein_isoform_2_[Homo_sapiens]; for *A. thaliana* PLP‐BP, https://bio‐web1.nscc‐gz.cn/job/result/44a9627d‐f166‐4e8b‐9f20‐bd3599a881f1/tr_Q944L8.

For each of the four proteins, a ConSurf analysis with default parameters was performed by providing the server with a multiple sequence alignment of homologous proteins from phylogenetically related organisms and the protein crystal structure, when available (YggS and yeast PLP‐BP; PDB codes: 1W8G and 1CT5, respectively), or alternatively its AlphaFold three‐dimensional model (human PROSC and plant PLP‐BP; O94903 PLPHP_HUMAN and Q944L8_ARATH, respectively). Concerning *E. coli* YggS, the BLASTP tool of the National Center for Biotechnology Information (Camacho et al., 2009) (https://blast.ncbi.nlm.nih.gov/Blast.cgi?PROGRAM=blastp&PAGE_TYPE=BlastSearch&LINK_LOC=blasthome) was used to retrieve homologous sequences by searching the experimental Clustered non‐redundant (*nr*) database, limited to the Bacteria kingdom and with the exclusion of the Pseudomonadota phylum. The search was performed using default parameters, allowing a maximum of 5000 target sequences. Retrieved representative sequences (*N* = 5000) were then filtered according to percentage of identity (within the 40%–100% range) and query coverage (98%–100% range), obtaining 586 sequences. A multiple alignment of these sequences was obtained by using the EMBL‐EBI Clustal Omega multiple sequence alignment program (Sievers et al., 2011) (https://www.ebi.ac.uk/jdispatcher/msa/clustalo). The multiple sequence alignment of human PROSC homologs (146 sequences) was obtained from a BLASTP search limited to the Eumetazoa subkingdom, followed by filtering of the retrieved representative sequences (1025) according to percentage of identity (within the 40%–100% range) and query coverage (93%–100% range). In the case of yeast PLP‐BP, the BLASTP search was limited to the Fungi kingdom and followed by filtering of the retrieved representative sequences (1691) according to percentage of identity (within the 40%–100% range) and query coverage (98%–100% range), obtaining 275 sequences. In the case of plant PLP‐BP, the BLASTP search was limited to the Angiospermae clade and followed by filtering of the retrieved representative sequences (716) according to percentage of identity (within the 40%–100% range) and query coverage (94%–100% range), obtaining 203 sequences.

### 4.13 Antibiotics susceptibility test

Antibiotic susceptibility was tested in BW25113 wild type and an *yggS E. coli* deletion strains (Babor et al., 2023; Prunetti et al., 2016) grown overnight in LB medium. Bacteria were collected, washed with normal saline solution and resuspended to match an OD_600_ of 1.5. Cell suspensions (100 μL) were mixed with 3 mL top agar (LB containing 6.5 g/L agar) and immediately plated on LB. Then, 6‐mm filter paper disks (Whatman No. 1), impregnated with 5 μL of 2, 4 and 8 μg/μL streptomycin, were placed on the surface of the agar. The zones of inhibition in mm were measured after 18 h. Experiments were performed in biological triplicates, and mean values with SEM are presented. For conducting streptomycin sensitivity assays in liquid media, overnight cultures were washed and resuspended in physiological solution. Growth curves of the indicated *E. coli* strains were obtained by measuring the optical density at 600 nm with a Microplate Reader (ThermoScientific Multiskan GO). The *E. coli* strains were grown in LB medium supplemented with streptomycin at the indicated concentrations. Each curve represents the average of three independent experiments.

## AUTHOR CONTRIBUTIONS

**Claudio Graziani:** Investigation; methodology; visualization; software. **Anna Barile:** Investigation; methodology; visualization; software. **Alessia Parroni:** Investigation; methodology. **Martino Luigi di Salvo:** Investigation; writing – review and editing. **Irene De Cecio:** Investigation; visualization. **Teresa Colombo:** Software; writing – review and editing; methodology. **Jill Babor:** Writing – review and editing. **Valérie de Crécy‐Lagard:** Writing – review and editing. **Roberto Contestabile:** Conceptualization; funding acquisition; writing – original draft; writing – review and editing; supervision; resources; validation; visualization; project administration; investigation; methodology; data curation. **Angela Tramonti:** Conceptualization; investigation; funding acquisition; writing – original draft; methodology; validation; visualization; writing – review and editing; project administration; data curation; supervision; resources.

## CONFLICT OF INTEREST STATEMENT

The authors declare that they have no conflicts of interests with the contents of this article.

## FINANCIAL STATEMENT

Open access publishing facilitated by Consiglio Nazionale delle Ricerche, as part of the Wiley ‐ CRUI‐CARE agreement.

## Supporting information


**FIGURE S1.** SDS‐PAGE and agarose gel electrophoresis analyses of protein fractions obtained from Size Exclusion Chromatography.
**FIGURE S2**. REMSA analyses showing the effect of poly‐His tag on RNA binding.
**FIGURE S3**. Control REMSA analysis with *e*SHMT and L‐TA.
**FIGURE S4**. Size exclusion chromatography analysis of purified recombinant PROSC.
**FIGURE S5**. Effect of DNase I (RNase free) and RNase on the nucleic acid copurified with PROSC.
**FIGURE S6**. REMSA analysis with purified apo‐YggS and total RNA extracted from either human or *E. coli* or cells.
**FIGURE S7**. Effect of DNase I (RNase free) and RNase on RNA captured by WT YggS.
**FIGURE S8**. Enrichment of *SsrA* and *RnpB* RNAs in the YggS‐bound and ‐unbound fractions, compared to total RNA, determined by RT‐qPCR.
**FIGURE S9**. Sequencing coverage from the cross‐linked samples and the non‐cross‐linked samples, for two selected regions of the *E. coli* BL21(DE3) genome.
**FIGURE S10**. Relative expression levels of *SsrA* and *RnpB* compared to genes that are involved in PLP metabolism in *E. coli* wild‐type (WT) and *yggS* deletion strains, determined by RT‐qPCR.
**FIGURE S11**. Results of the ConSurf analysis of PLP‐Bs from four different sources.
**FIGURE S12**. Superimposed three‐dimensional models of PLP‐BPs from different sources.
**FIGURE S13**. Prediction of disordered protein regions of PLP‐BP from different sources.
**FIGURE S14**. Cryo‐EM structure of accommodated trans‐translation complex on *E. coli* stalled ribosome (PDB ID: 7ac7).
**FIGURE S15**. Sensitivity to streptomycin of WT and *yggS E. coli* strains.

**TABLE S1.** Aligned reads resulting from the RNA‐seq of RNA captured by WT and K36A YggS.

**TABLE S2.** Differential analysis of RNA‐seq results obtained from RNA samples copurified with cross‐linked and non‐cross‐linked WT YggS.
