# Supplementary material for: The ubiquitous pyridoxal 5′‐phosphate‐binding protein is also an RNA‐binding protein
Source: Protein Sci. 2024 Nov 27;33(12):e5242. doi: 10.1002/pro.5242 (PMC11602438; doi:10.1002/pro.5242)
Supplement: Supplementary file 1 — FIGURE S1. SDS‐PAGE and agarose gel electrophoresis analyses of protein fractions obtained from Size Exclusion Chromatography. FIGURE S2. REMSA analyses showing the effect of poly‐His tag on RNA binding. FIGURE S3. Control REMSA analysis with eSHMT and L‐TA. FIGURE S4. Size exclusion chromatography analysis of purified recombinant PROSC. FIGURE S5. Effect of DNase I (RNase free) and RNase on the nucleic acid copurified with PROSC. FIGURE S6. REMSA analysis with purified apo‐YggS and total RNA extracted from either human or E. coli or cells. FIGURE S7. Effect of DNase I (RNase free) and RNase on RNA captured by WT YggS. FIGURE S8. Enrichment of SsrA and RnpB RNAs in the YggS‐bound and ‐unbound fractions, compared to total RNA, determined by RT‐qPCR. FIGURE S9. Sequencing coverage from the cross‐linked samples and the non‐cross‐linked samples, for two selected regions of the E. coli BL21(DE3) genome. FIGURE S10. Relative expression levels of SsrA and RnpB compared to genes that are involved in PLP metabolism in E. coli wild‐type (WT) and yggS deletion strains, determined by RT‐qPCR. FIGURE S11. Results of the ConSurf analysis of PLP‐Bs from four different sources. FIGURE S12. Superimposed three‐dimensional models of PLP‐BPs from different sources. FIGURE S13. Prediction of disordered protein regions of PLP‐BP from different sources. FIGURE S14. Cryo‐EM structure of accommodated trans‐translation complex on E. coli stalled ribosome (PDB ID: 7ac7). FIGURE S15. Sensitivity to streptomycin of WT and yggS E. coli strains. [file PRO-33-e5242-s001.pdf]

Fig. S1

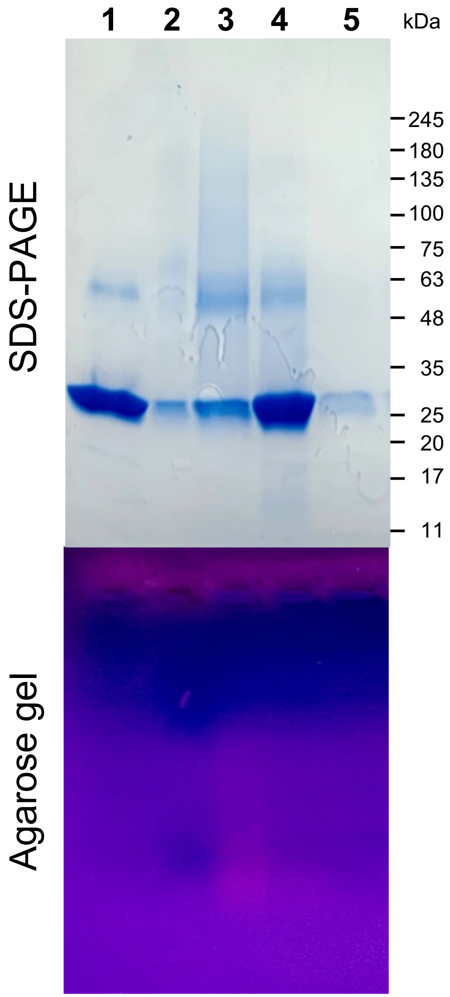

#	Elution volume (ml)	sample
1	16-20	not crosslinked
2	8-10	crosslinked
3	12-16	
4	16-20	
5	20-22	

Figure S1. SDS-PAGE (upper panel) and agarose gel electrophoresis (lower panel) analyses of protein fractions obtained from Size Exclusion Chromatography of cross-linked and untreated YggS shown in Fig. 2B. On top of the main protein band corresponding to YggS, a minor higher molecular weight band is also visible in all samples, which may correspond to a dimeric form of the protein but is not evident from the SEC analysis (Fig. 2B). Nucleic acid is clearly visible only in lane 3 of the agarose gel, which correspond to the large size form of YggS that is visible in the elution profile of crossed-linked YggS shown in Fig. 2B (elution volume 12-16 mL).

Fig. S2

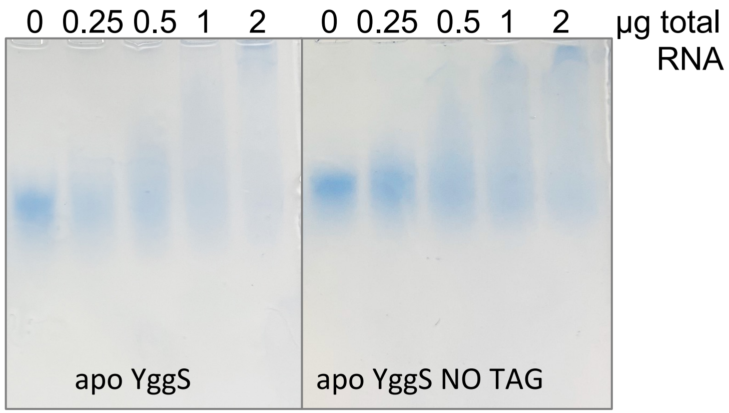

Figure S2. REMSA analyses carried out with purified apo-YggS (1 µg) with or without the poly-histidine tag incubated with increasing amounts (0.25, 0.5, 1 and 2 µg) of total RNA extracted from BW25113 *E. coli* cells.

Fig. S3

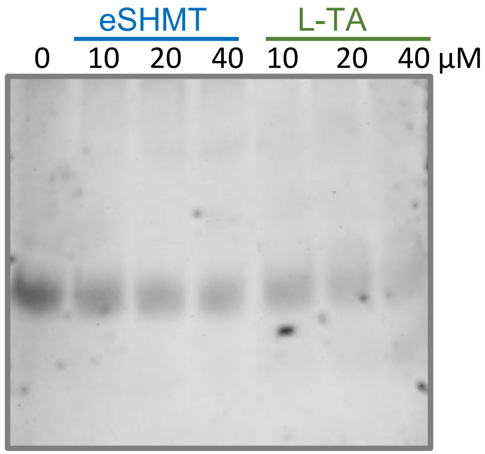

Figure S3. Control REMSA analysis using a fixed amount of *E. coli* tRNA (20 ng) and the indicated increasing concentrations of *e*SHMT and L-TA.

Fig. S4

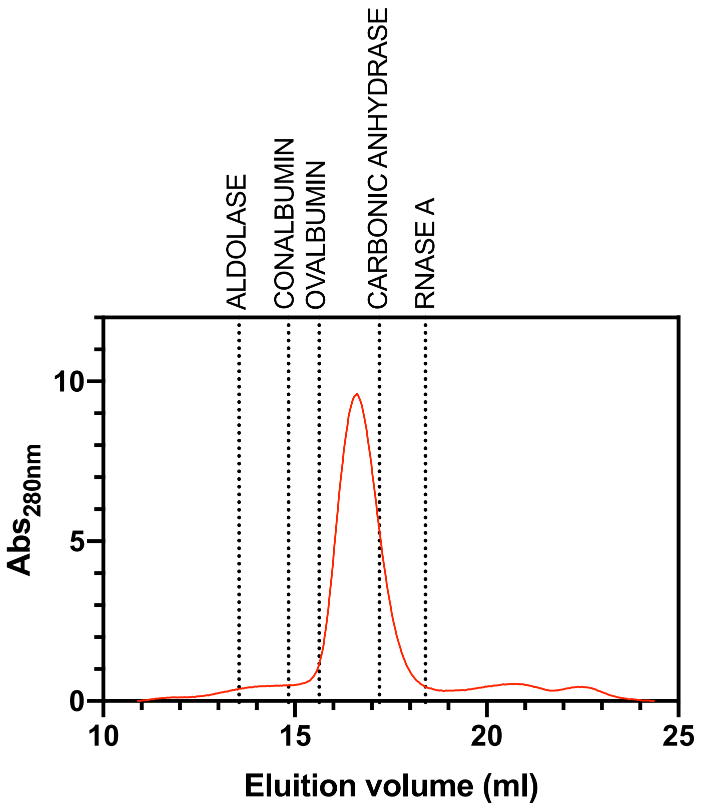

Figure S4. Size Exclusion Chromatography analysis of purified recombinant PROSC. The main elution band centred at an elution volume of 16.5 mL corresponds to the monomeric form of the protein. A minor elution band is also visible with lower elution volume (around 14 mL) corresponding to the dimeric form of the protein.

Fig. S5

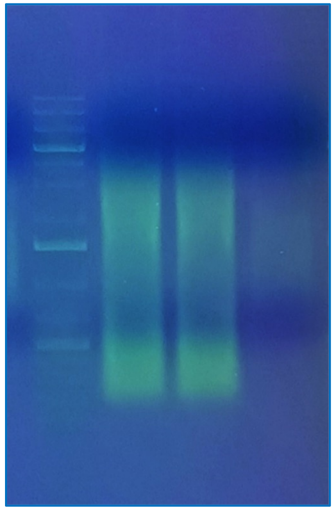

Figure S5. Agarose gel electrophoresis of PROSC fraction 2 shown in Fig. 8A untreated (un) and after incubation with either DNase I (RNase free) or RNase I. The 1Kb plus DNA ladder was used as molecular weight standard (Thermo Scientific).

Fig. S6

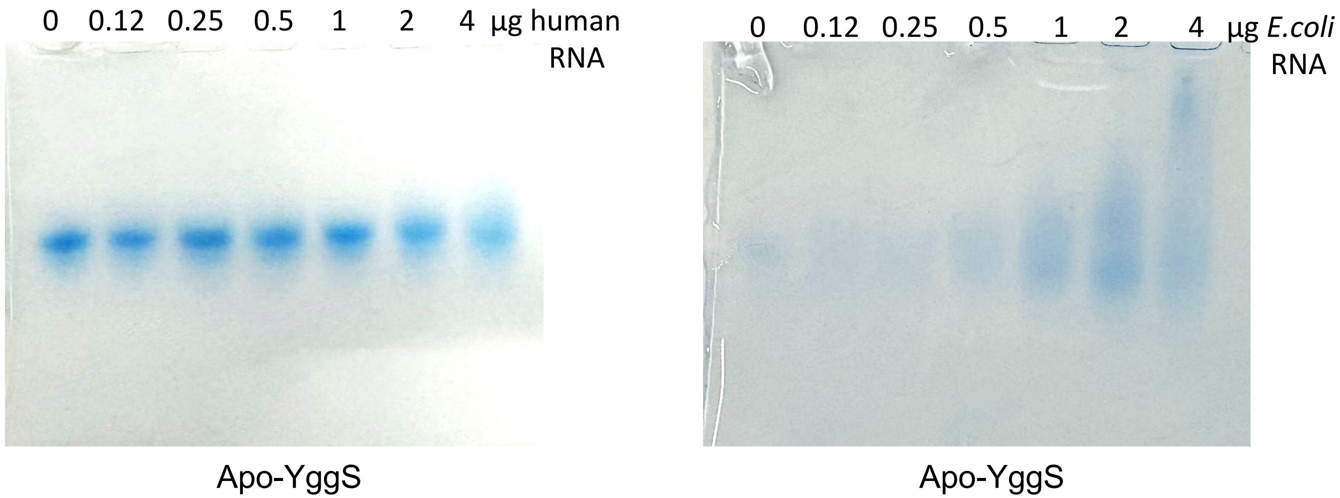

Figure S6. REMSA analysis carried out with purified apo-YggS (1 µg) and total RNA extracted from either *E. coli* or human cells.

Fig. S7

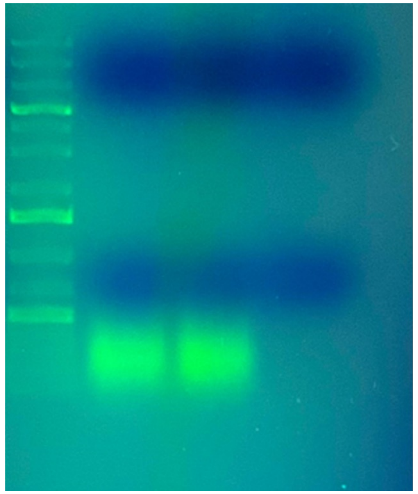

Figure S7. Agarose gel electrophoresis analysis of nucleic acid retained in the RNA capture experiments which used WT YggS as bait (lane marked with "E" in Fig. 9A) untreated (un) and treated with either DNAse I (RNase free) or RNase.

Fig. S8

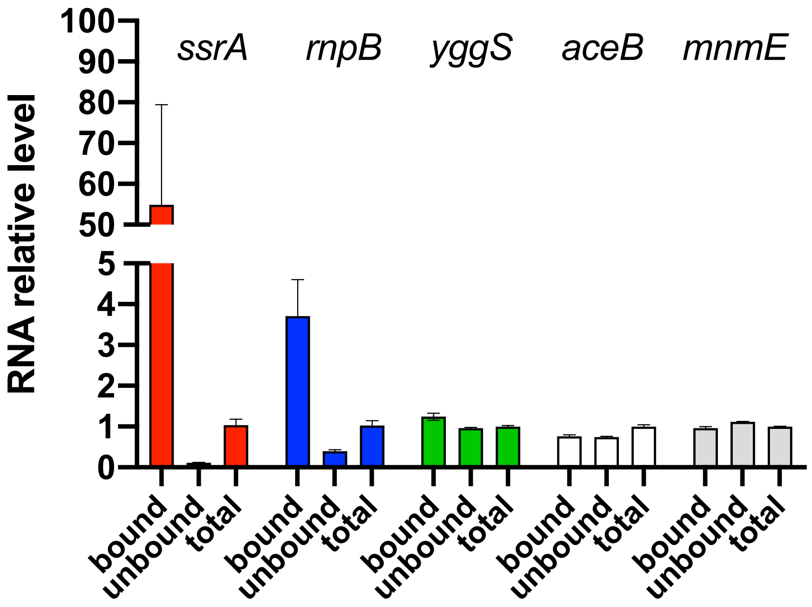

Figure S8. Enrichment of *SsrA* and *RnpB* compared to *YggS*, *AceB* and *MnmE* RNAs in the YggS-bound and -unbound fractions, compared to total RNA, determined by RT-qPCR.

Fig. S9

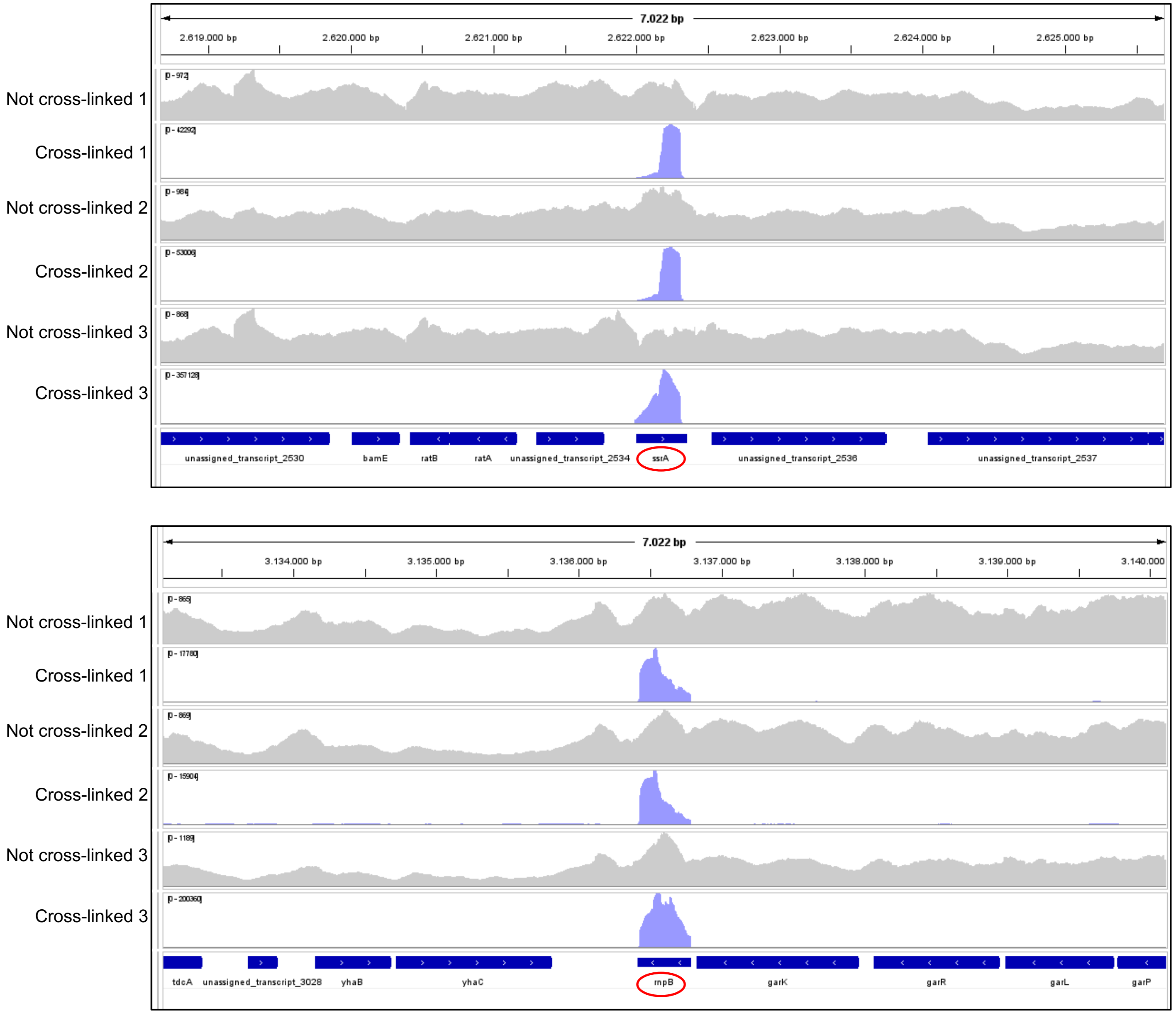

Figure S9. Sequencing coverage from the cross-linked samples (in blue) and the non-cross-linked samples (in grey), for two selected regions of the *E. coli* BL21(DE3) genome. The first region contains the *ssrA* gene (upper panel), while the other one includes the *rnpB* gene (lower panel). The values in brackets indicate the total number of reads aligning to the genome within the visualized region. Coverage for both genes is significantly and specifically higher for the cross-linked samples than for the non cross-linked samples.

Fig. S10

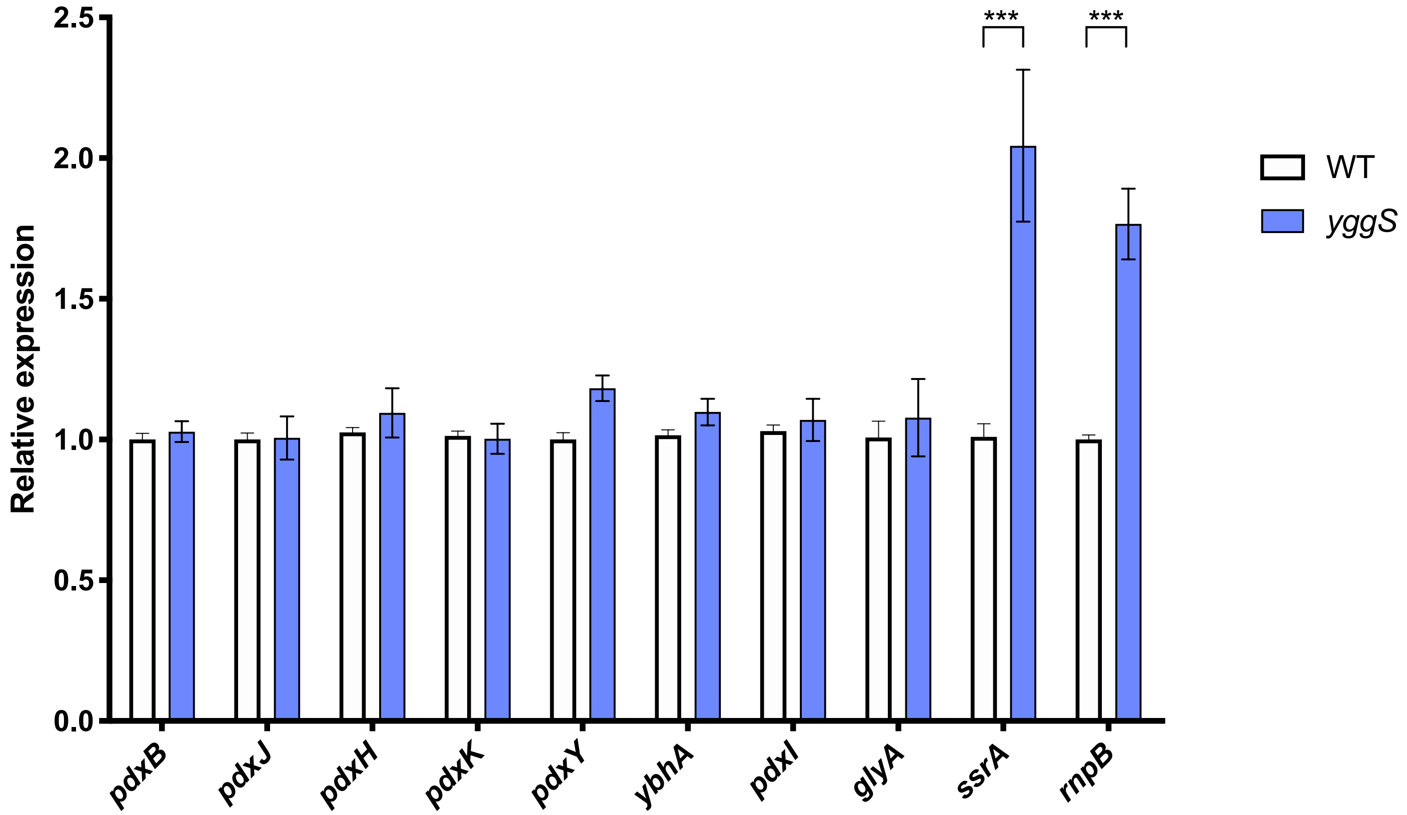

Figure S10. Relative expression levels of *ssrA* and *rnpB* compared to genes that are involved in PLP metabolism in *E. coli* wild-type (WT) and *yggS* deletion strains, determined by RT-qPCR. Bacteria were grown in M9 minimal medium to exponential phase. The level of expression of each gene in *yggS* deletion strains was referred to expression in the wild-type strain. Experimental values are reported as the mean ± SEM. Statistical significance was determined using Student's t-test. P values are <0.0005 (***).

Fig. S11

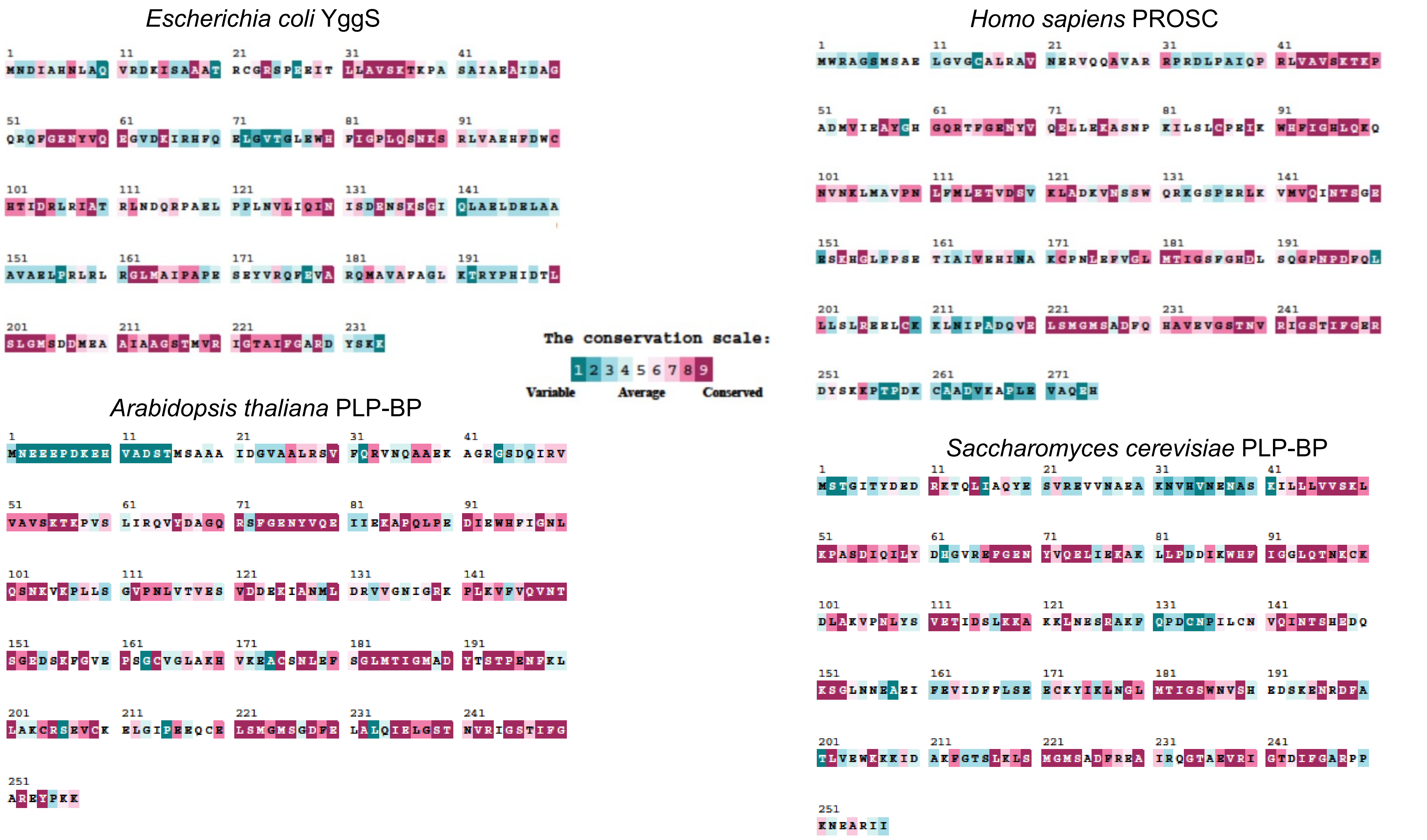

Figure S11. Results of the ConSurf analysis of PLP-BPs from four different sources carried out as explained in the text. Amino acid residues are coloured according to the indicated conservation scale.

Fig. S12

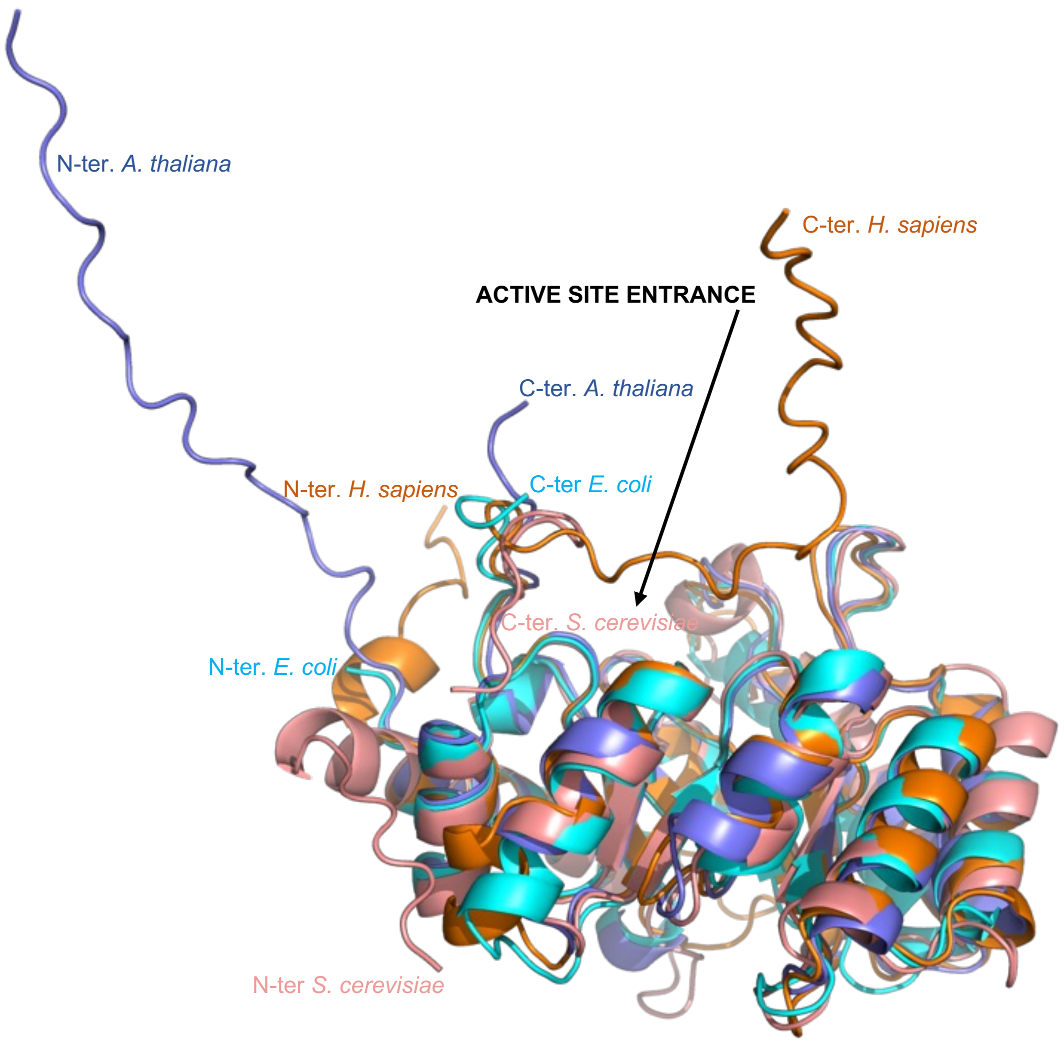

Figure S12. Superimposed three-dimensional models of PLP-BPs from *E. coli* (cyan), *H. sapiens* (orange), *S. cerevisiae* (salmon) and *A. thaliana* (slate) obtained from the AlphaFold Protein Structure Database (https://alphafold.ebi.ac.uk/; PLPHP_HUMAN; Q944L8_ARATH, YGGS and PLPHP_YEAST). The C- and N-terminal ends of the proteins are labelled in the corresponding colours. The PLP-binding site entrance is also indicated by a black arrow.

Fig. S13

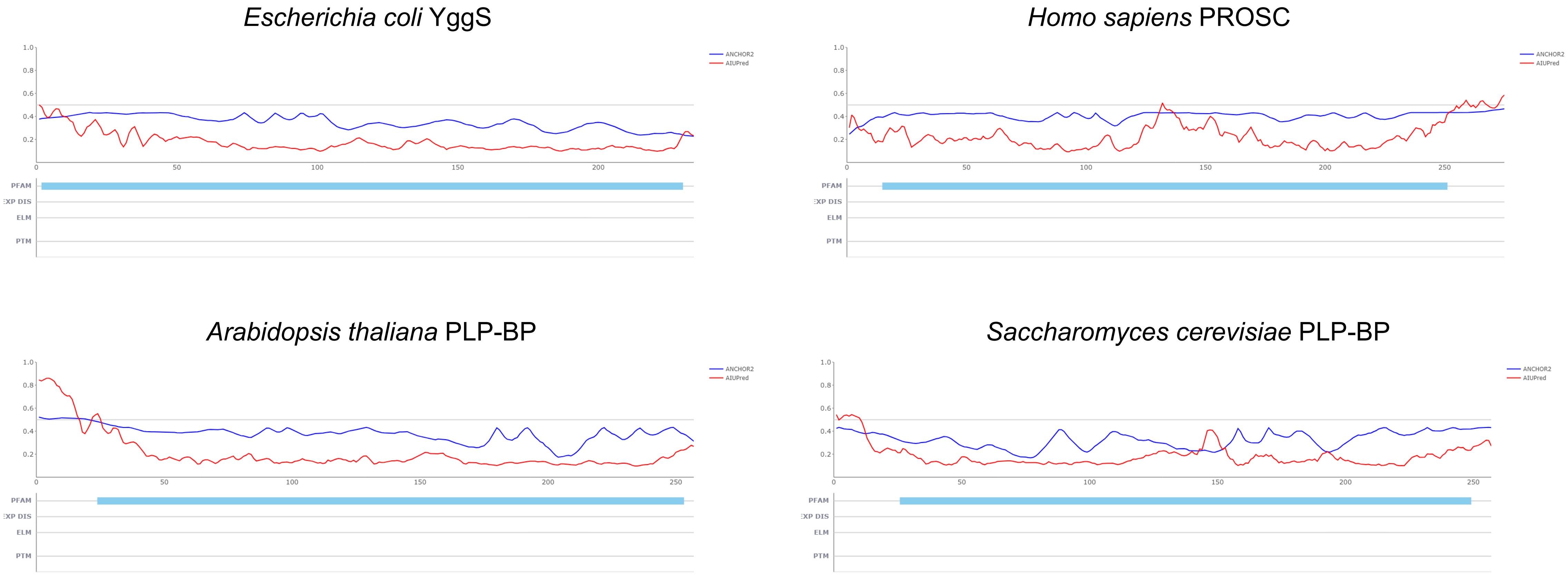

Figure S13. Prediction of disordered protein regions of PLP-BPs obtained using the AIUPred web interface (https://iupred.elte.hu/) and protein regions that do or do not adopt a stable structure depending on ligand binding, as predicted by AIUPred using the ANCHOR2.

Fig. S14

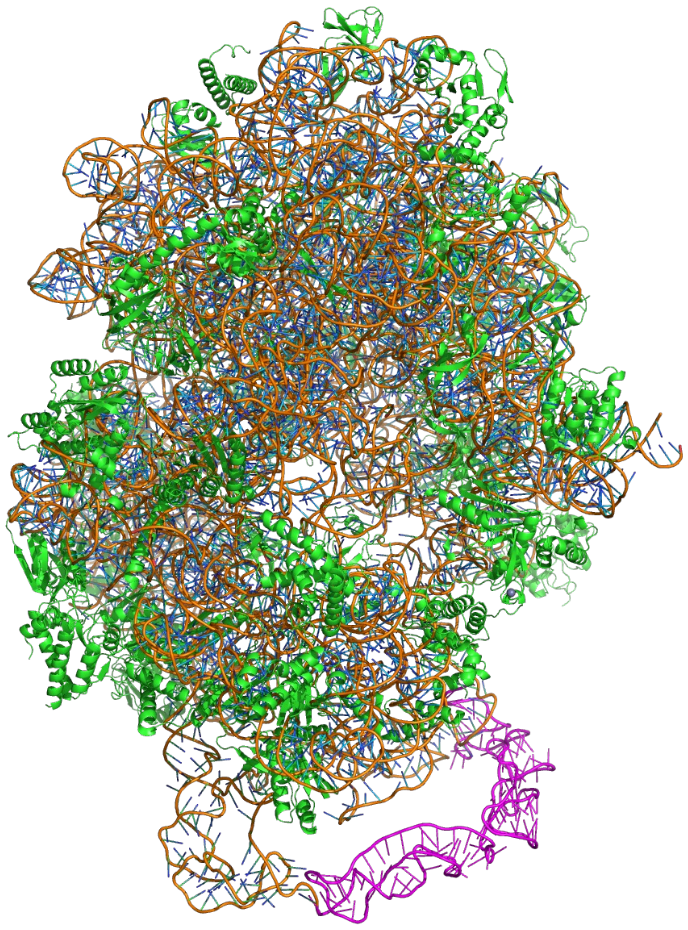

Figure S14. Cryo-EM structure of accomodated trans-translation complex on *E. coli* stalled ribosome (PDB ID: 7ac7). The region of *SsrA* encompassing positions 198 and 318 of the nucleotide sequence is coloured in magenta.

Fig. S15

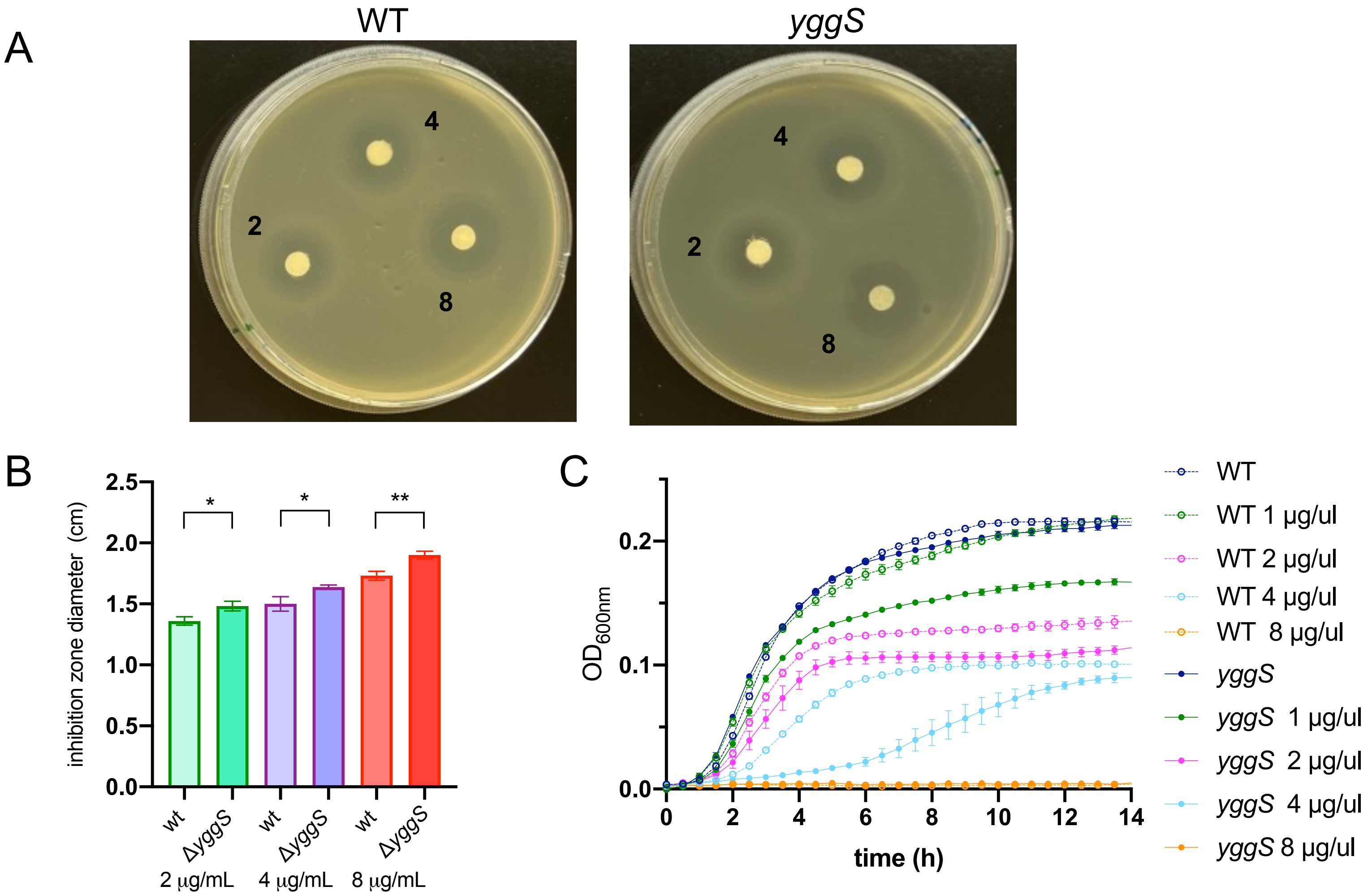

Figure S15. Sensitivity to streptomycin of WT and *yggS* deletion *E. coli* strains. A) Inhibition zone assays performed on solid rich medium (LB). Plated bacterial cells (OD_{600} = 0.006) were treated with 5 µl of 2, 4 and 8 µg/µl streptomycin deposited on paper discs, as indicated by numbers on the figure. All assays were repeated three times. B) reports the average ± SEM of the measured inhibition diameter. Statistical significance was determined using the Student's t-test. P-values are < 0.005 (**) and < 0.05 (*). C) shows the growth curves of wild-type and *yggS* strains obtained by measuring the optical density at 600 nm. The *E. coli* strains were grown in LB liquid medium supplemented with streptomycin at the indicated concentrations. Each curve represents the average ± SEM of three independent experiments.
